# Supplementary material for: A building-block design for enhanced visible-light switching of diarylethenes
Source: Nat Commun. 2019 Sep 17;10:4232. doi: 10.1038/s41467-019-12302-6 (PMC6748945; doi:10.1038/s41467-019-12302-6)
Supplement: Supplementary file 1 — Supplementary Information [file 41467_2019_12302_MOESM1_ESM.pdf]

Supplementary Information

A building-block design for enhanced visible-light switching of diarylethenes

Zhiwei Zhang,[1] Wenhui Wang,[1] Peipei Jin,[2] Jiadan Xue,[2] Lu Sun,[3] Jinhai Huang,[1] Junji Zhang,[1*] He Tian[1]

[1]Key Laboratory for Advanced Materials and Joint International Research Laboratory of Precision Chemistry and Molecular Engineering, Feringa Nobel Prize Scientist Joint Research Center, School of Chemistry and Molecular Engineering, East China University of Science & Technology, 130 Meilong Road, Shanghai, 200237, China

[2]Department of Chemistry, Zhejiang Sci-Tech University, Hangzhou, 310018, China

[3]Institute of Modern Optics, Nankai University, Tianjin 300071, China

Email addresses: zhangjunji@ecust.edu.cn (J. Z.)

Supplementary Figures

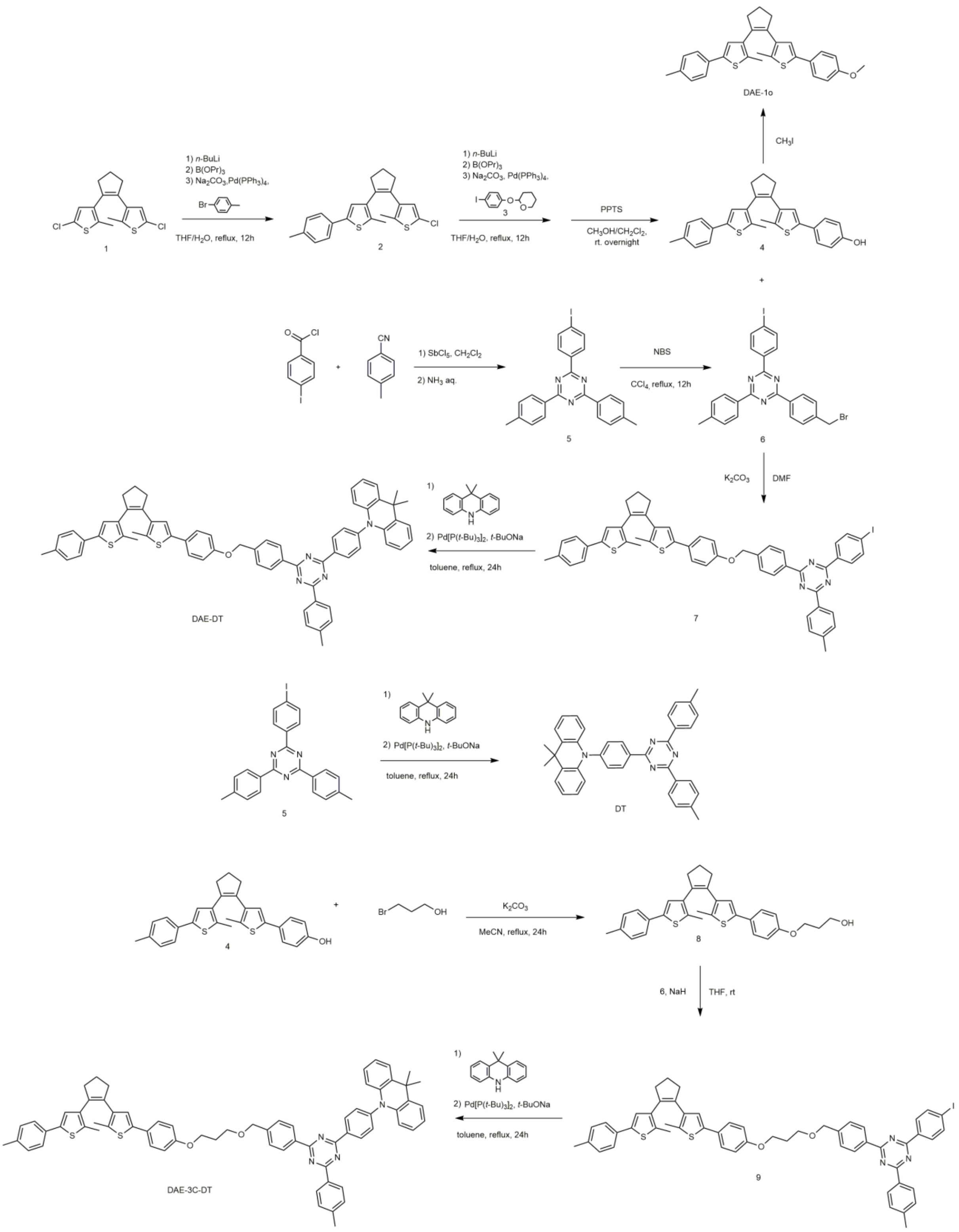

Supplementary Figure 1. Route for the synthesis of the investigated compounds.

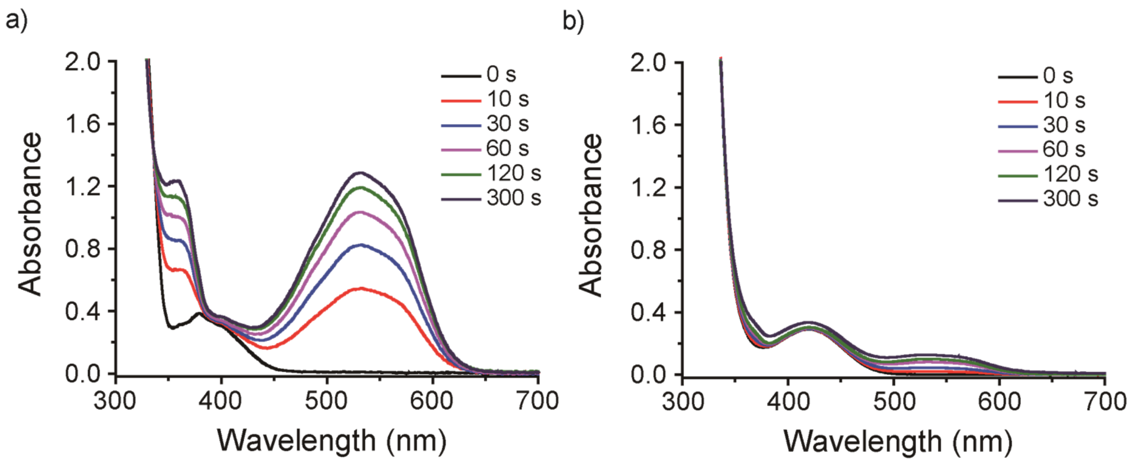

Supplementary Figure 2. Absorption spectra of a) DAE-1o/DT and b) DAE-1o/PT in deaerated toluene upon irradiation at 420 nm, respectively. [DAE-1o] = [DT] = [PT] = 10^{-4} M.

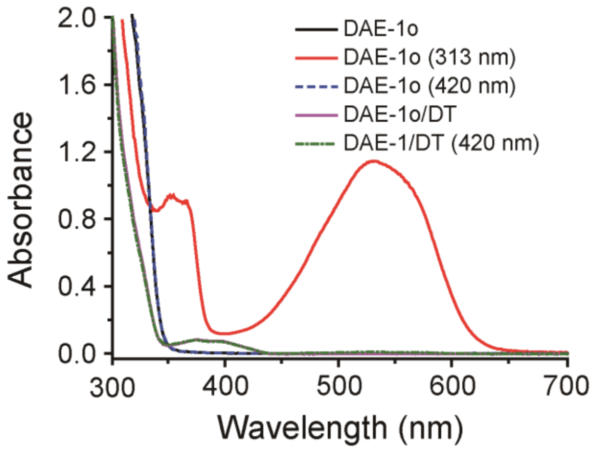

Supplementary Figure 3. Absorption spectra of DAE-1o (1.0×10^{-4} M) in toluene upon irradiation at 313 nm and 420 nm, DAE-1o/DT (2.0×10^{-5} M/2.0×10^{-5} M) in deaerated toluene before and after irradiation at 420 nm.

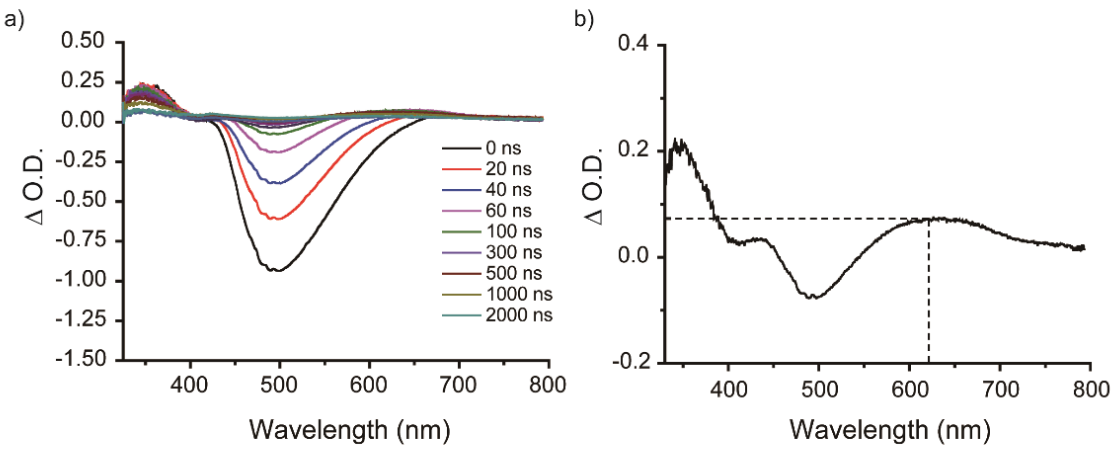

Supplementary Figure 4. Nanosecond time-resolved transient absorption spectra of DT (2.5×10^{-4} M, deaerated toluene) a) at different decay times; b) at 100 ns.

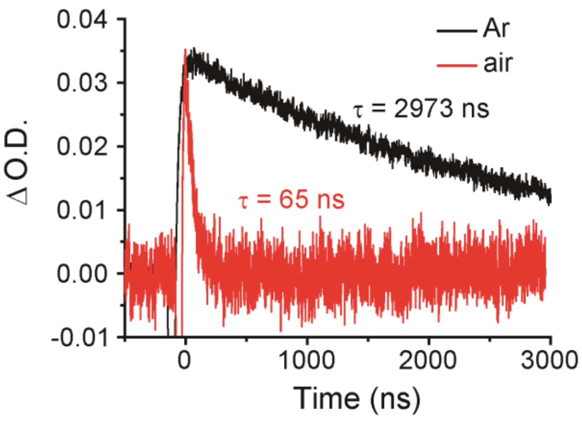

Supplementary Figure 5. Transient absorption decays of DT (1.0 $\times10^{-4}$ M) at 620 nm in deaerated (black) and aerated (red) toluene, respectively.

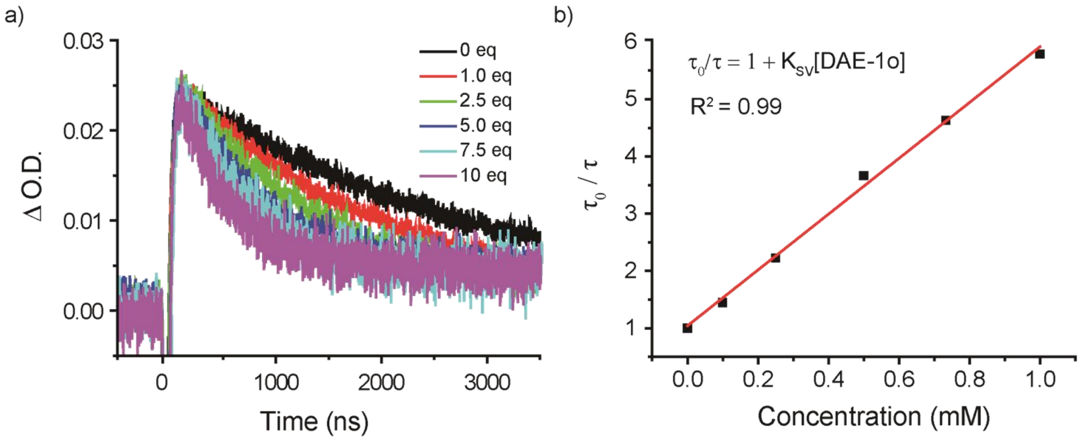

Supplementary Figure 6. a) Transient absorption decays of DT (1.0×10^{-4} M) at 620 nm by adding different equivalents of DAE-1o in deaerated toluene; b) Stern-Volmer plot and linear fit for quenching of DT by DAE-1o.

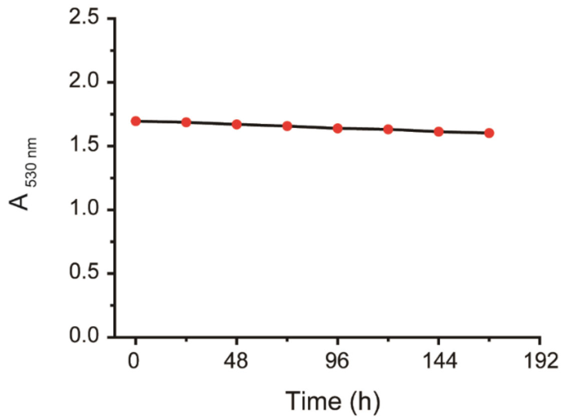

Supplementary Figure 7. The absorption decay of PSS for DAE-DT (1.0 $\times 10^{-4}$ M) monitored at 530 nm at room temperature in the dark in deaerated toluene.

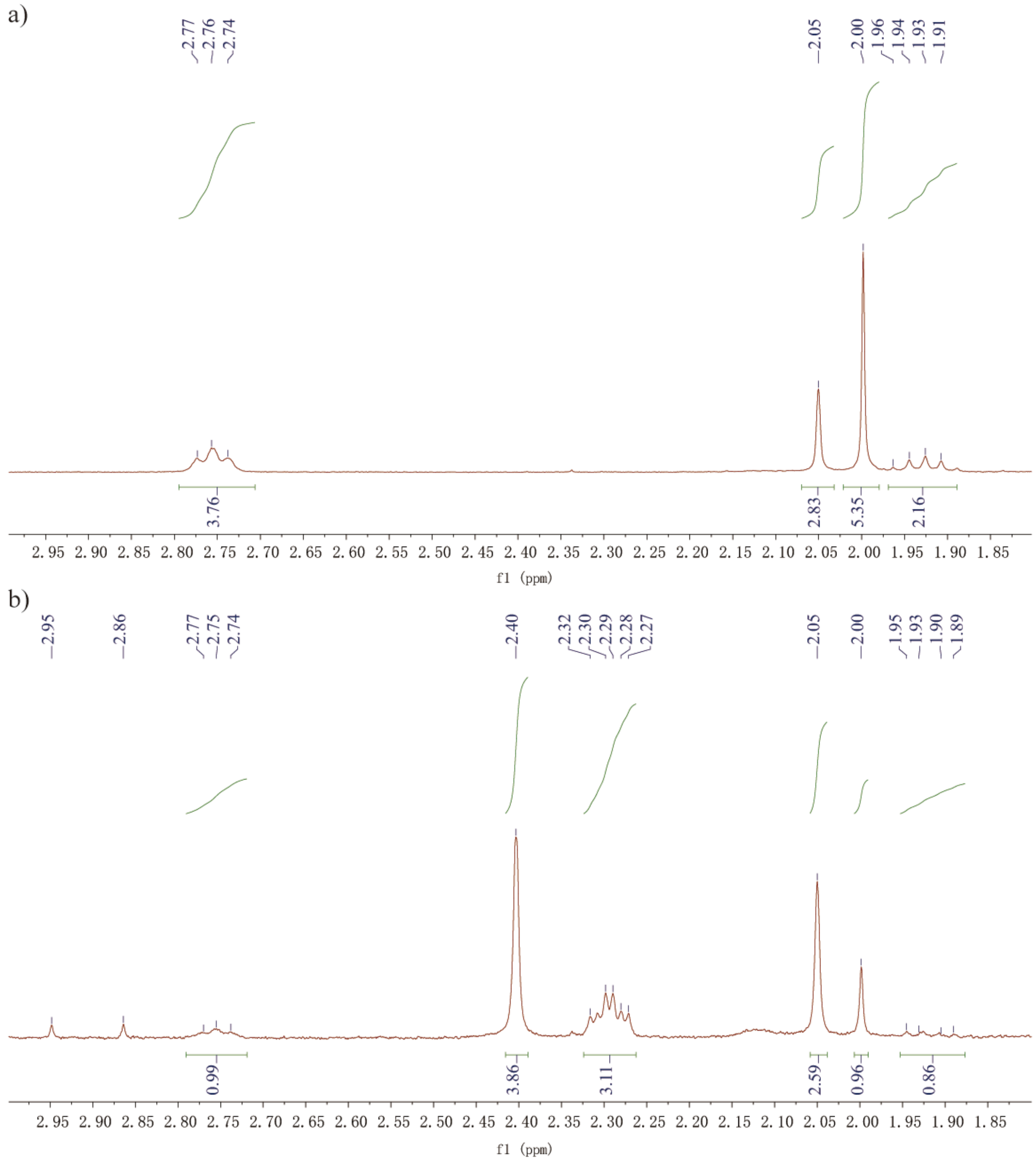

Supplementary Figure 8. ^{1}H NMR spectra of DAE-1o (3.1 $\times 10^{-3}$ M) (a) before and (b) after photoirradiation at 313 nm in [D$_6$]benzene.

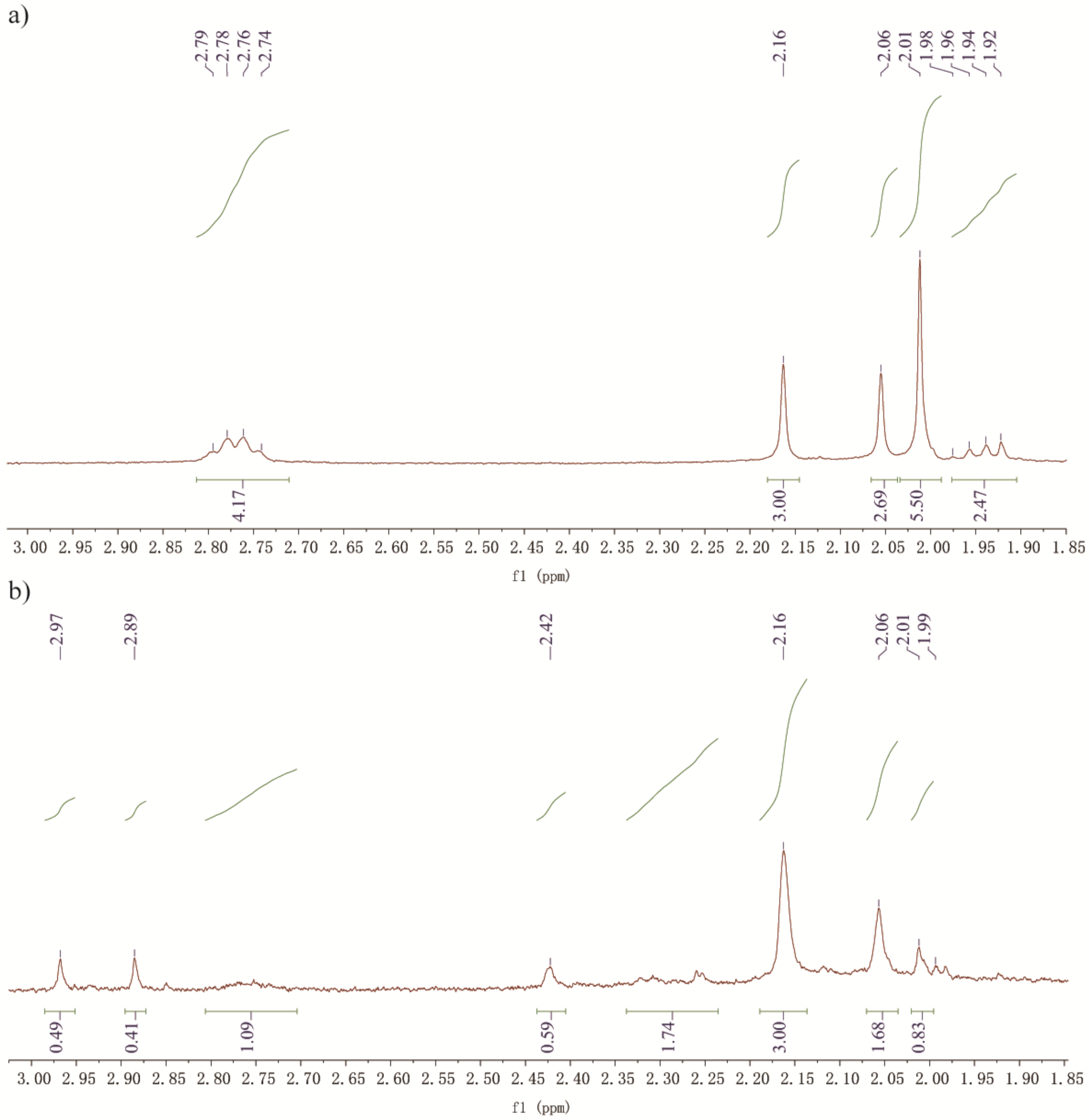

Supplementary Figure 9 ^{1}H NMR spectra of DAE-o-DT (3.1×10^{-3} M) (a) before and (b) after photoirradiation at 420 nm in [D_6]benzene.

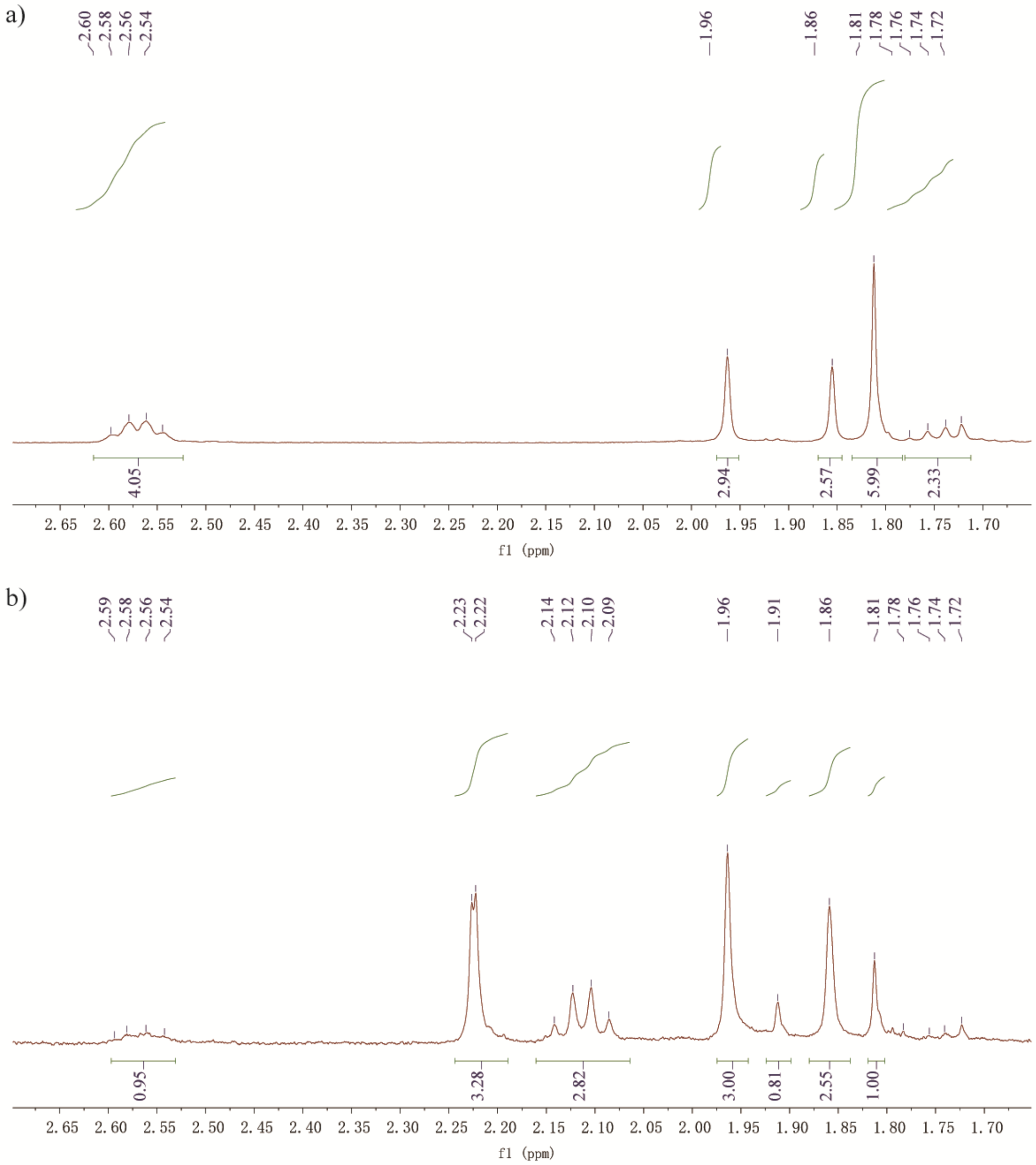

Supplementary Figure 10. ^{1}H NMR spectra of DAE-o-DT (3.1 × 10^{-3} M) (a) before and (b) after photoirradiation at 420 nm in deaerated [D$_6$]benzene.

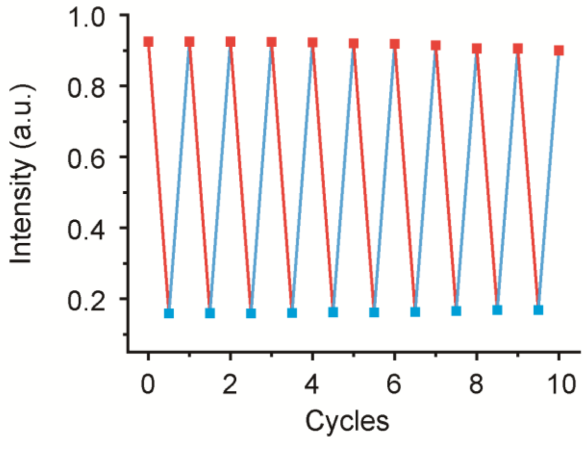

Supplementary Figure 11. Emission of DAE-DT (2.0 $\times 10^{-5}$ M) at 495 nm in deaerated toluene during repetitive switching cycles consisting of alternate 420 nm/> 550 nm irradiations.

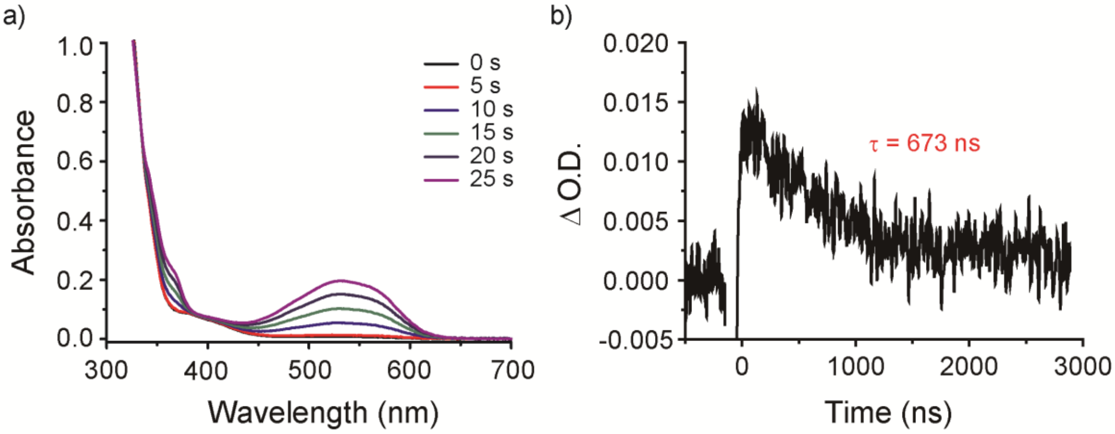

Supplementary Figure 12. a) The photochromic performance of DAE-3C-DT (2.0×10^{-5} M) under irradiation at 420 nm at different times; b) Transient absorption decays of DAE-3C-DT (1.0×10^{-4} M) at 620 nm in deaerated toluene.

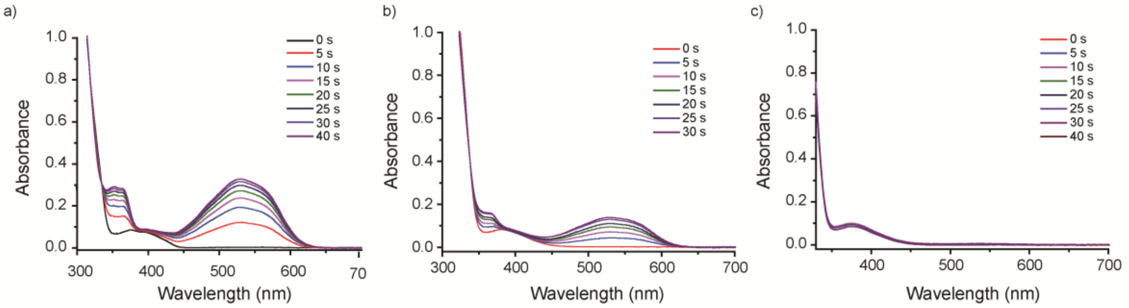

Supplementary Figure 13. The absorbance spectra of DAE-o-DT (2.0 $\times 10^{-5}$ M) upon irradiation at 420 nm in deaerated solvents of a) cyclohexane; b) chloroform; c) acetone.

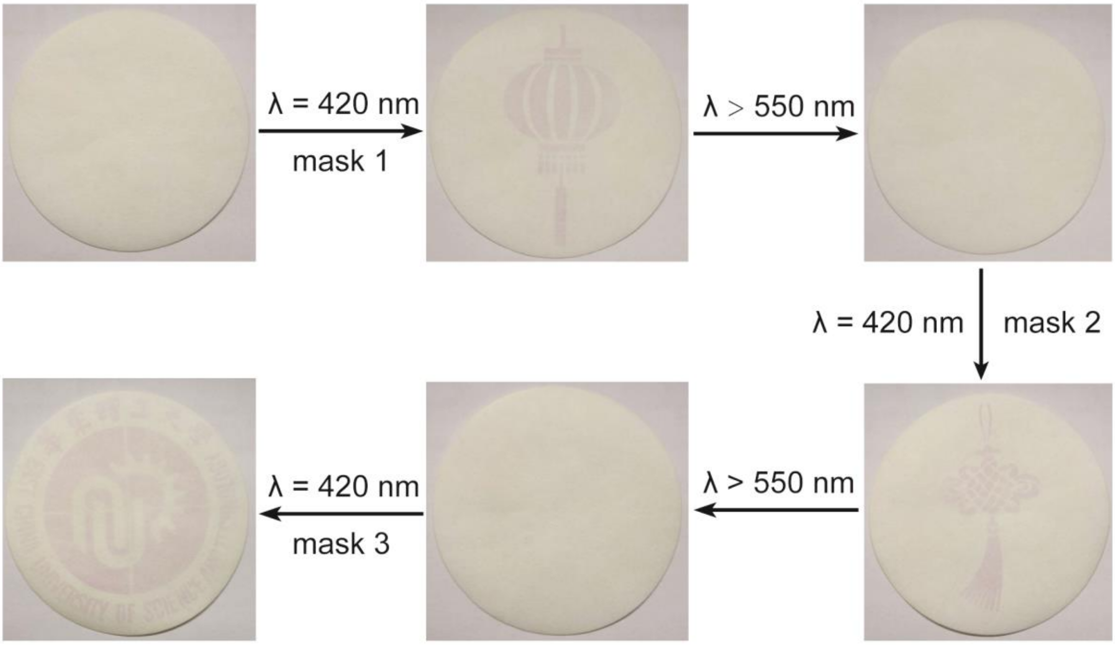

Supplementary Figure 14. A series of images (Chinese lantern, Chinese knot and the badge of East China University of Science and Technology) were sequentially written onto and erased from the same filter paper with DAE/DT using different masks with 420 nm irradiation for 7 min and > 550 nm irradiation for 10 min, respectively.

Supplementary Tables

Supplementary Table 1. The value of singlet/triplet (S_1/T_1) excited state energy for investigated compounds.

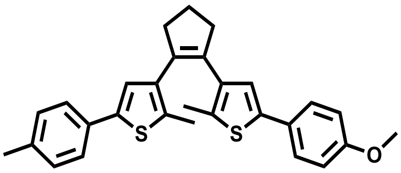 DAE-1o	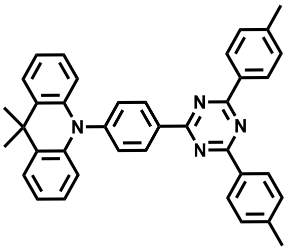 DT
Calculated: S_1 = 4.1876 eV T_1 = 2.4911 eV	Calculated: S_1 = 2.5356 eV T_1 = 2.5280 eV

Supplementary Table 2. Computational photocyclization conversion ratios of the given switch isomers at the depicted wavelengths (λ_{irr}) in degassed toluene. [DAE-1o] = [DAE-o-DT] = 2×10^{-5} M.

Photoreaction	λ_{irr} (nm)	Conversion ratios（PSS）
DAE-1o→DAE-1c	313	85%
DAE-o-DT→DAE-c-DT	313	81%
DAE-o-DT→DAE-c-DT	420	80%

Supplementary Table 3. Experimental photocyclization conversion ratios of the given switch isomers at the depicted wavelengths (λ_{irr}) in degassed toluene by ^{1}H NMR.

Photoreaction	λ_{irr} (nm)	Conversion ratios（PSS）
DAE-1o→DAE-1c	313	80%
DAE-o-DT→DAE-c-DT	313	85%
DAE-o-DT→DAE-c-DT	420	77%

Supplementary Table 4. Photocyclization quantum yields of DAE-o-DT at 420 nm in different degassed solvents. [DAE-o-DT] = 2×10^{-5} M.

Solvents	φ
cyclohexane	0.40
chloroform	0.26
acetone	0.008

Supplementary Table 5. DFT calculations of low-energy excitation transition for investigated compounds.

Compound	Excitation	Coefficient	Contribution	Strength
DAE-1o Singlet	HOMO→LUMO	0.68902	0.9495	0.0595
DAE-1o triplet	HOMO→LUMO	0.38829	0.3015	
	HOMO→LUMO+1	-0.35358	0.2500	
	HOMO-1→LUMO	0.29945	0.1793	
	HOMO-1→LUMO+1	0.24058	0.1158	
DT singlet	HOMO→LUMO	0.69964	0.978992	0.0001
DT triplet	HOMO→LUMO	0.69895	0.977062	

Supplementary Table 6. DFT calculations of energy values of investigated compounds.

Compound	Orbital	Energy (hartree)	Energy (eV)
DAE-1o	HOMO-1	-0.1988	-5.40935
	HOMO	-0.18665	-5.07875
	LUMO	-0.03119	-0.84868
	LUMO+1	-0.02540	-0.69113
DAE-1c	HOMO-1	-0.20328	-5.53125
	HOMO	-0.15712	-4.27524
	LUMO	-0.06692	-1.82089
	LUMO+1	-0.02405	-0.65440
DAE-o-DT	HOMO-1	-0.17963	-4.88773
	HOMO	-0.17962	-4.88746
	LUMO	-0.07229	-1.96701
	LUMO+1	-0.07225	-1.96592
DAE-c-DT	HOMO-1	-0.17975	-4.891
	HOMO	-0.15859	-4.31523
	LUMO	-0.07275	-1.97953
	LUMO +1	-0.07262	-1.97599
DT	HOMO-1	-0.23345	-6.35217
	HOMO	-0.18177	-4.94596
	LUMO	-0.06938	-1.88783
	LUMO +1	-0.06624	-1.80239

Supplementary Methods

Theoretical calculations

Density functional theory (DFT) calculations were employed to optimize the ground state geometries of the molecules, using the B3LYP functional[1] and the 6-31G(d) basis set[2]. At the optimized geometries, time-dependent DFT (TDDFT) calculations were carried out using the 6-311+ G(d,p) basis set[3]. All calculations were carried out using the Gaussian09 program package[4].

Photoreaction quantum yields measurements

The photoreaction quantum yields of DAEs at 313 nm, 420 nm and 546 nm were measured with the potassium ferrioxalate ($K_3[Fe(C_2O_4)_3]$) as the actinometer[5-6]. The solutions are prepared as follows: In the dark, 3.0 mL of a 0.006 M (for 313 nm and 420 nm) or 0.15 M (for 546 nm) solution of potassium ferrioxalate in 0.05 M H_2SO_4 in the cuvette (1 cm quartz cell) is irradiated at the investigated wavelength for 5 min. Subsequently, 0.5 mL of phenanthroline (0.1 wt% in 0.5 M H_2SO_4/1.6 M NaOAc) were added in the cuvette and the absorbance at 510 nm was measured immediately. The irradiation time must be short in order to avoid more than 10% ferrioxalate decomposition. The moles of Fe^{2+} could be calculated *via* formula Equation (1):

$$\text{moles } Fe^{2+} = \frac{V_1 \times \Delta A(510\ nm)}{V_2 \times l \times \varepsilon(510\ nm)} \quad (1)$$

Where, V_2 = 3.0 mL, which is the irradiated volume, V_1 = 3.5 mL, which is the final volume after complexation with phenanthroline. l = 1 cm, which is the optical pathlength of the irradiation cell. ε (510 nm) is 11100 M^{-1} cm^{-1}, which is the molar extinction coefficient of the complex $Fe(phen)_3^{2+}$. ΔA is the optical difference in absorbance between the irradiated solution and that taken in the dark. Thus, the moles of photons absorbed by the irradiated solution per time unit are as follows:

$$\frac{Nhv}{t} = \frac{moles\ of\ Fe^{2+}}{\varphi_\lambda \times t \times F} \quad (2)$$

Where φ_λ is the quantum yield of ferrous ion production at the irradiation wavelength, t is the time of irradiation, and F is the mean fraction of light absorbed by DAEs solution. φ_λ is the quantum yield at the used irradiation wavelength (1.24 for 313 nm, 1.12 for 420 nm and 0.15 for 546 nm).

Then, 3 mL of solution of DAE-1o or DAE-1o/DT or DAE-o-DT in solvents in a cuvette was irradiated by the same investigated wavelength in the dark and the absorbance at 530 nm was tested immediately. The concentration of DAE-1o, DAE-o-DT are both 2.0 $\times 10^{-5}$ M. The photocyclization quantum yields can be calculated in following formula Equation (3):

$$\varphi_x = \frac{\Delta A/\Delta t}{(Nhv/t) \times \varepsilon_x \times F_x} \tag{3}$$

Where, $\Delta A/\Delta t$ is the change of absorbance upon irradiation at detective wavelength, ε_x is the molar extinction coefficient at detective wavelength ($\varepsilon_{530\ nm}$ = 17906 M^{-1} cm^{-1} for DAE-1c, 19000 M^{-1} cm^{-1} for DAE-c-DT, determined in Section 2) and F_x is the mean fraction of light absorbed, the value of which is $1\text{-}10^{-A}$.

For the photocycloreversion quantum yield, 3 mL of solution of DAE-1c or DAE-c-DT in deaerated toluene in a cuvette was irradiated by 546 nm light in the dark and the absorbance at 530 nm was tested immediately. The concentration of DAE-1c, DAE-c-DT are both 2.0 $\times 10^{-5}$ M. The photocycloreversion quantum yields can be calculated in following formula Equation (4):

$$\varphi_x = -\frac{\Delta A/\Delta t}{(Nhv/t) \times \varepsilon_x \times F_x} \tag{4}$$

Where, $\Delta A/\Delta t$ is the change of absorbance upon irradiation at detective wavelength, ε_x is the molar extinction coefficient at detective wavelength and F_x is the mean fraction of light absorbed, the value of which is $1\text{-}10^{-A}$.

Synthesis of compounds

Compound 2[7]

Compound 1 (1.0 g, 3.0 mmol) was dissolved in 10 mL of dry THF and cooled to -78 ℃ under argon. To this solution *n*-BuLi (2.2 M in hexane, 1.4 mL, 3.0 mmol) was slowly added and the mixture was stirred for 1 h at this temperature. Then $B(OPr)_3$ (1.2 mL, 3.9 mmol) was dropped into the solution and stirred for 1 h at room temperature. 4-Bromotoluene (1.1 g, 3.6 mmol) was dissolved in 25 mL of degassed THF under argon. $Pd(PPh_3)_4$ (360 mg, 0.31 mmol) was added to the second solution followed by addition of 20 mL of degassed aqueous of 3.5 M sodium carbonate solution. The first solution was added to the second without further purification and the mixture was refluxed for 12 h. The aqueous phase was extracted with DCM and the organic phase was washed with saturated NaCl aqueous, then dried over $MgSO_4$ and evaporated under reduced pressure. The crude product was purified by column chromatography

on silica gel (PE) to obtain 2 after drying in vacuo as colorless solids. (795 mg, 69%).^{1}H NMR (400 MHz, $CDCl_3$) δ 7.39 (d, *J* = 8.1 Hz, 2H), 7.15 (d, *J* = 8.0 Hz, 2H), 6.94 (s, 2H), 2.91 - 2.65 (m, 4H), 2.34 (s, 3H), 2.04 (dt, *J* = 14.9, 7.5 Hz, 2H), 1.98 (s, 3H), 1.87 (s, 3H). ^{13}C NMR (101 MHz, $CDCl_3$) δ 139.75, 139.48, 138.43, 136.82, 136.65, 136.59, 134.66, 134.60, 133.96, 133.48, 131.81, 129.50, 127.64, 126.83, 125.27, 123.55, 123.01, 117.91, 115.74, 38.53, 23.07, 21.18, 14.46, 14.41. HRMS (ESI) (m/z): $[M+H]^+$ calcd for $[C_{22}H_{22}ClS_2]^+$, 385.0846; Found, 385.0840.

Compound 4

Compound 2 (384 mg, 1.0 mmol) was dissolved in 5 mL of dry THF and cooled to -5 ℃ under argon. To this solution *n*-BuLi (2.2 M in hexane, 0.7 mL, 1.5 mmol) was slowly added and the mixture was stirred for 1 h at this temperature. Then $B(OPr)_3$ (0.6 mL, 1.9 mmol) was dropped into the solution and stirred for 1 h at room temperature. 3 (360 mg, 1.2 mmol) was dissolved in 25 mL of degassed THF under argon. $Pd(PPh_3)_4$ (180 mg, 0.15 mmol) was added to the second solution followed by addition of 20 mL of degassed aqueous of 3.5 M sodium carbonate solution. The first solution was added to the second without further purification and the mixture was refluxed for 12 h. The aqueous phase was extracted with DCM and the organic phase was washed with saturated NaCl aqueous, then dried over $MgSO_4$ and evaporated under reduced pressure to obtain the crude products. The solution of the crude products (526 mg) and pyridinium *p*-toluenesulfonate (PPTS) (20 mg, 0.08mmol) in $MeOH/CH_2Cl_2$ (20 mL) was stirred at room temperature overnight. The reaction mixture was concentrated under reduced pressure, and the residue was purified by chromatography on silica gel (PE:EA = 5:1) to give 5 (248 mg, 56%). ^{1}H NMR (400 MHz, $CDCl_3$) δ 7.39 (t, *J* = 8.5 Hz, 4H), 7.14 (d, *J* = 7.5 Hz, 2H), 7.00 (s, 1H), 6.91 (s, 1H), 6.80 (d, *J* = 7.2 Hz, 2H), 2.84 (t, *J* = 7.3 Hz, 4H), 2.35 (s, 3H), 2.12-2.03 (m, 2H), 1.98 (d, *J* = 2.9 Hz, 6H). ^{13}C NMR (101 MHz, $CDCl_3$) δ 155.61, 154.84, 139.75, 139.48, 138.43, 136.82, 136.65, 136.59, 134.66, 134.60, 133.96, 133.48, 131.81, 129.50, 127.64, 126.83, 125.27, 123.55, 123.01, 117.91, 115.74, 38.53, 29.76, 23.07, 21.18, 14.46, 14.41. HRMS (ESI) (m/z): $[M+H]^-$ calcd for $[C_{28}H_{25}OS_2]^-$, 441.1347; found, 441.1345.

DAE-1o

The solution of 4 (221 mg, 0.5 mmol), CH_3I (142 mg, 1.00 mmol), and anhydrous acetone (30 mL) was stirring for 24 h at 41 ℃ under argon. Then the resulting mixture was cooled to room temperature and quenched with saturated aqueous NH_4Cl. After that, the residue was extracted by ethyl acetate, and then dried over $MgSO_4$. After removing solvent under reduced pressure, the residue was purified by chromatography on silica gel (PE:EA = 10:1) to obtain

DAE-1o (160 mg, 70%). ^{1}H NMR (400 MHz, $CDCl_3$) δ 7.36-7.19 (m, 4H), 7.07 (d, *J* = 7.9 Hz, 2H), 6.92 (s, 1H), 6.84 (s, 1H), 6.81 (s, 1H), 6.79 (s, 1H), 3.74 (s, 3H), 2.76 (t, *J* = 7.5 Hz, 4H), 2.27 (s, 3H), 2.00 (dt, *J* = 14.8, 7.5 Hz, 2H), 1.91 (d, *J* = 3.1 Hz, 6H). ^{13}C NMR (101 MHz, $CDCl_3$) δ 158.83, 139.73, 139.51, 136.77, 136.62, 136.56, 134.65, 134.57, 133.94, 133.47, 131.81, 129.46, 127.52, 126.60, 125.26, 124.40, 123.51, 122.99, 114.21, 55.39, 38.50, 35.01, 34.47, 31.96, 31.54, 30.17, 29.74, 29.40, 23.05, 22.73, 21.14, 14.39, 14.16. HRMS (ESI) (m/z): $[M+Na]^+$ calcd for $[C_{29}H_{28}OS_2Na]^+$, 479.1479; found, 479.1473.

Compound 5

A mixture of 4-iodobenzoyl chloride (2.92 g, 11.0 mmol) and 4-Methylbenzonitrile (2.57 g, 22.0 mmol) in 15 mL of dichloromethane was cooled to -5 ℃ and stirred for 30 min, followed by adding antimony chloride (3.30 g, 11.0 mmol) dropwise to the solution. The mixture was stirred for 1 h at room temperature and then refluxed overnight. The cooled mixture was filtered and the obtained yellow solid was washed with dichloromethane. The solid was slowly added to 75 mL of 28% ammonia solution at -5 ℃ and kept stirring for 30 min at this temperature. Subsequently, the mixture was stirred for 3 h at room temperature. Then, the mixture was filtered, and the collected white solid was washed with plenty of water. The solid was added to 30 mL of *N*, *N'*-dimethylformamide and stirred at 160 ℃ for 30 min. The insoluble solid was separated by filtration. The solvent was removed under vacuum to obtain 6 (3.82 g, 75%) as a white solids. This material was used in the next step without further purification. ^{1}H NMR (400 MHz, $CDCl_3$) δ 8.64 (d, *J* = 8.2 Hz, 6H), 7.69 (d, *J* = 8.6 Hz, 2H), 7.37 (d, *J* = 8.0 Hz, 4H), 2.48 (s, 6H). ^{13}C NMR (101 MHz, $CDCl_3$) δ 171.58, 170.79, 143.17, 137.84, 133.44, 130.44, 129.40, 128.96, 99.84, 22.72, 21.77, 14.15. HRMS (ESI) (m/z): $[M+H]^+$ calcd for $[C_{23}H_{19}IN_3]^+$, 464.0624; found, 464.0627.

Compound 6

The solution of 5 (83 mg, 0.18 mmol), *N*-Bromosuccinimide (7.74 mg, 0.18 mmol) and Benzoyl peroxide (12.2 mg, 0.05 mmol) in CCl_4 (5 mL) was refluxed overnight. After cooling to the room temperature, the reaction mixture was concentrated under reduced pressure, and the residue was purified by chromatography on silica gel (PE:DCM = 20:1) to give pure 6 (53 mg, 54%) as white solids. ^{1}H NMR (400 MHz, $CDCl_3$) δ 8.64 (d, *J* = 8.3 Hz, 2H), 8.56 (d, *J* = 8.1 Hz, 2H), 8.40 (d, *J* = 8.5 Hz, 2H), 7.85 (d, *J* = 8.4 Hz, 2H), 7.52 (d, *J* = 8.2 Hz, 2H), 7.31 (d, *J* = 8.0 Hz, 2H), 4.52 (s, 2H), 2.42 (s, 3H).^{13}C NMR (101 MHz, $CDCl_3$) δ 170.99, 143.44, 142.15,

137.92, 136.23, 135.81, 133.22, 130.45, 129.48, 129.42, 129.34, 129.00, 100.08, 32.76, 21.80. HRMS (ESI) (m/z): $[M+H]^+$ calcd for $[C_{23}H_{18}N_3BrI]^+$, 541.9729; found, 541.9731.

Compound 7

The solution of 6 (540 mg, 1.00 mmol), 4 (484 mg, 1.10 mmol), K_2CO_3 (210 mg, 1.50 mmol), NaI (4.5 mg, 0.03 mmol) in anhydrous DMF (50 mL) was stirred at 110 ^oC for 24 h under argon. After cooling to the room temperature, the reaction mixture was concentrated under reduced pressure, and the residue was dissolved in CH_2Cl_2 (20 mL). The organic solution was washed with H_2O (100 mL) and brine (100 mL) and dried over $MgSO_4$. The solven was concentrated under reduced pressure and crude product was purified by chromatography on silica gel (PE:DCM = 3:1) to give pure 7 (551 mg, 61%) as white solids. ^1H NMR (400 MHz, $CDCl_3$) δ 8.66 (d, *J* = 8.2 Hz, 2H), 8.55 (d, *J* = 8.2 Hz, 2H), 8.38 (d, *J* = 8.5 Hz, 2H), 7.83 (d, *J* = 8.5 Hz, 2H), 7.53 (d, *J* = 8.2 Hz, 2H), 7.39 - 7.25 (m, 6H), 7.06 (d, *J* = 8.0 Hz, 2H), 6.93 - 6.87 (m, 4H), 5.12 (s, 2H), 2.76 (t, *J* = 7.4 Hz, 4H), 2.40 (s, 3H), 2.26 (s, 3H), 2.06 - 1.95 (m, 2H), 1.90 (d, *J* = 1.5 Hz, 6H). ^{13}C NMR (101 MHz, $CDCl_3$) δ 170.66, 170.21, 169.87, 156.74, 142.30, 140.62, 138.69, 138.32, 136.85, 135.72, 135.55, 134.84, 134.73, 133.57, 132.88, 132.56, 132.25, 130.75, 129.41, 128.41, 128.20, 127.96, 126.93, 126.26, 125.61, 124.20, 122.46, 122.08, 114.21, 98.96, 68.63, 37.44, 30.91, 28.68, 21.99, 21.67, 20.74, 20.10, 13.35. HRMS (ESI) (m/z): $[M+H]^+$ calcd for $[C_{51}H_{43}IN_3OS_2]^+$, 904.1892; found, 904.1915.

DT

The solution of 6 (463 mg, 1.00 mmol), 9,9-Dimethyl-9,10-dihydroacridine (230 mg, 1.10 mmol), *t*-BuONa (47 mg, 0.50 mmol), Pd[P(*t*-Bu_3)]$_2$ (25 mg, 0.05 mmol) and anhydrous toluene (50 mL) was stirred and refluxed for two days under argon. The cooled mixture was partitioned between CH_2Cl_2 and water. The organic layer was separated, and the aqueous layer was extracted with chloroform. The combined organic layers were washed with brine, dried over $MgSO_4$ and concentrated under reduced pressure. The residue was purified by chromatography on silica gel (PE:DCM = 3:1) to afford pure DT (408 mg, 75%) as yellow-green solids. ^1H NMR (400 MHz, $CDCl_3$) δ 9.01 (d, *J* = 8.3 Hz, 2H), 8.70 (d, *J* = 8.1 Hz, 4H), 7.55 (d, *J* = 8.2 Hz, 2H), 5.51 (d, J = 7.6 Hz, 2H), 7.49 (dd, *J* = 7.4, 1.8 Hz, 4H), 7.03 - 6.90 (m, 4H), 6.40 - 6.36 (m, 2H), 2.50 (s, 6H), 1.73 (s , 6H). ^{13}C NMR (101 MHz, $CDCl_3$) δ 170.69, 142.22, 139.60, 135.31, 132.45, 130.52, 130.45, 129.16, 128.43, 127.98, 125.42, 124.31, 119.77, 113.16, 35.01, 30.29, 20.75. HRMS (ESI) (m/z): $[M+H]^+$ calcd for $[C_{38}H_{33}N_4]^+$, 545.2705; found, 545.2696.

DAE-o-DT

The solution of 7 (200 mg, 0.22 mmol), 9,9-Dimethyl-9,10-dihydroacridine (69 mg, 0.33 mmol), *t*-BuONa (47 mg, 0.50 mmol), Pd[P(*t*-Bu)$_3$]$_2$ (10 mg, 0.02 mmol) and anhydrous toluene (40 mL) was stirred and refluxed for 24 h under argon. The cooled mixture was partitioned between CH_2Cl_2 and water. The organic layer was separated, and the aqueous layer was extracted with chloroform. The combined organic layers were washed with brine, dried over $MgSO_4$ and concentrated under reduced pressure. The residue was purified by column chromatography on silica gel (PE:DCM = 2:1) to afford pure DAE-o-DT (173 mg, 80%) as yellow-green solids. ^{1}H NMR (400 MHz, $CDCl_3$) δ 8.94 (d, *J* = 8.5 Hz, 2H), 8.75 (d, *J* = 8.3 Hz, 2H), 8.63 (d, *J* = 8.2 Hz, 2H), 7.58 (d, *J* = 8.3 Hz, 2H), 7.49 (d, *J* = 8.5 Hz, 2H), 7.42 (dd, *J* = 7.5, 1.8 Hz, 2H), 7.39-7.29 (m, 6H), 7.06 (d, *J* = 8.0 Hz, 2H), 6.92-6.85 (m, 8H), 6.31 (dd, *J* = 7.9, 1.5 Hz, 2H), 5.16 (s, 2H), 2.76 (t, *J* = 7.4 Hz, 4H), 2.43 (s, 3H), 2.26 (s, 3H), 2.05-1.97 (m, 2H), 1.91 (d, *J* = 1.8 Hz, 6H), 1.66 (s, 6H). ^{13}C NMR (101 MHz, $CDCl_3$) δ 172.07, 171.62, 171.20, 158.00, 145.49, 143.61, 141.95, 140.84, 139.94, 139.57, 136.96, 136.79, 136.39, 136.06, 134.81, 134.13, 133.81, 133.57, 132.00, 131.79, 131.71, 130.46, 129.71, 129.65, 129.51, 129.26, 128.20, 127.58, 126.86, 126.66, 125.56, 125.45, 123.71, 123.34, 121.06, 115.47, 114.42, 69.92, 38.68, 36.27, 31.52, 29.92, 27.43, 25.75, 23.24, 22.00, 21.34, 14.61. HRMS (ESI) (m/z): $[M+H]^+$ calcd for $[C_{66}H_{57}N_4OS_2]^+$, 985.3974; found, 985.3979.

Compound 8

The solution of 4 (75 mg, 0.170 mmol), 3-Bromo-1-propanol (74 mg, 0.509 mmol), K_2CO_3 (70 mg, 0.509 mmol), in anhydrous MeCN (30 mL) was stirred at 80 ^{o}C for 24 h under argon. After cooling to the room temperature, the reaction mixture was concentrated under reduced pressure, and the residue was dissolved in CH_2Cl_2 (20 mL). The organic solution was washed with H_2O (100 mL) and brine (100 mL) and dried over $MgSO_4$. The solven was concentrated under reduced pressure and crude product was purified by chromatography on silica gel (PE:DCM = 1:4) to give pure 8 (80.0 mg, 94%) as white solids. ^{1}H NMR (400 MHz, $CDCl_3$) δ 7.40 (t, *J* = 7.9 Hz, 4H), 7.14 (d, *J* = 7.9 Hz, 2H), 6.99 (s, 1H), 6.91 (s, 1H), 6.87 (d, *J* = 8.7 Hz, 2H), 4.13 (t, *J* = 5.9 Hz, 2H), 3.87 (t, *J* = 5.9 Hz, 2H), 2.83 (t, *J* = 7.4 Hz, 4H), 2.34 (s, 3H), 2.11 - 2.01 (m, 4H), 1.98 (d, *J* = 3.0 Hz, 6H). HRMS (ESI) (m/z): $[M+H]^+$ calcd for $[C_{51}H_{43}IN_3OS_2]^+$, 523.1741; found, 523.1741.

Compound 9

The solution of 8 (60 mg, 0.120 mmol) in anhydrous THF (20 mL) was stirred at 0 °C for 5 min under argon. Then NaH (60% in mineral oil, 24 mg, 0.600 mmol) was added to the solution and was stirred at 0 °C for 30 min under argon. The solution of 6 (136 mg, 0.25 mmol) in anhydrous THF was added to the mentioned solution via a needle. The reaction mixture was then stirred at room temperature for 6 h. The reaction mixture was quenched with deionized water (10 mL) at 0 ℃ and the organic solution was washed with H_2O (100 mL) and brine (100 mL) and dried over $MgSO_4$. The solven was concentrated under reduced pressure and crude product was purified by chromatography on silica gel (PE:THF = 10:1) to give pure 9 (22.8 mg, 20%) as white solids. ^{1}H NMR (400 MHz, $CDCl_3$) δ 8.69 (d, *J* = 8.3 Hz, 2H), 8.63 (d, *J* = 8.2 Hz, 2H), 8.46 (d, *J* = 8.5 Hz, 2H), 7.90 (d, *J* = 8.5 Hz, 2H), 7.51 (d, *J* = 8.3 Hz, 2H), 7.38 (dd, *J* = 16.7, 8.5 Hz, 6H), 7.12 (d, *J* = 8.0 Hz, 2H), 6.98 (s, 1H), 6.90 (s, 1H), 6.87 (d, *J* = 8.8 Hz, 2H), 4.65 (s, 2H), 4.12 (t, *J* = 6.2 Hz, 2H), 3.72 (t, *J* = 6.1 Hz, 2H), 2.83 (t, *J* = 7.4 Hz, 4H), 2.47 (s, 3H), 2.32 (s, 3H), 2.13 (m, 2H), 2.06 (m, 2H), 1.97 (d, *J* = 4.0 Hz, 6H). HRMS (ESI) (m/z): $[M+Na]^+$ calcd for $[C_{54}H_{48}IN_3O_2S_2Na]^+$, 984.2130; found, 984.2131.

DAE-3C-DT

The solution of 9 (100 mg, 0.10 mmol), 9,9-Dimethyl-9,10-dihydroacridine (33 mg, 0.16 mmol), *t*-BuONa (24 mg, 0.25 mmol), Pd[P(*t*-Bu)$_3$]$_2$ (10 mg, 0.02 mmol) and anhydrous toluene (20 mL) was stirred and refluxed for 24 h under argon. The cooled mixture was partitioned between CH_2Cl_2 and water. The organic layer was separated, and the aqueous layer was extracted with chloroform. The combined organic layers were washed with brine, dried over $MgSO_4$ and concentrated under reduced pressure. The residue was purified by column chromatography on silica gel (PE:THF = 10:1) to afford pure DAE-3C-DT (78 mg, 72%) as yellow-green solids. ^{1}H NMR (400 MHz, $CDCl_3$) δ 9.01 (d, *J* = 8.5 Hz, 2H), 8.77 (d, *J* = 8.3 Hz, 2H), 8.70 (d, *J* = 8.2 Hz, 2H), 7.56 (d, *J* = 2.9 Hz, 2H), 7.54 (d, *J* = 2.7 Hz, 2H), 7.50 (d, *J* = 1.7 Hz, 1H), 7.48 (d, *J* = 1.9 Hz, 1H), 7.42 – 7.36 (m, 6H), 7.12 (d, *J* = 8.1 Hz, 2H), 7.02 - 6.92 (m, 7H), 6.87 (s, 1H), 6.38 (dd, *J* = 7.8, 1.6 Hz, 2H), 4.66 (s, 2H), 4.13 (t, *J* = 6.2 Hz, 2H), 3.74 (t, *J* = 6.1 Hz, 2H), 2.82 (t, *J* = 7.4 Hz, 4H), 2.49 (s, 3H), 2.32 (s, 3H), 2.14 (m, *J* = 6.1 Hz, 2H), 2.10 – 2.00 (m, 2H), 1.96 (d, *J* = 8.8 Hz, 6H), 1.73 (s, 6H). HRMS (ESI) (m/z): $[M+H]^+$ calcd for $[C_{69}H_{63}N_4O2S_2]^+$, 1043.4392; found, 1043.4388.

Characterization data

4 (^{1}H NMR, $CDCl_3$)

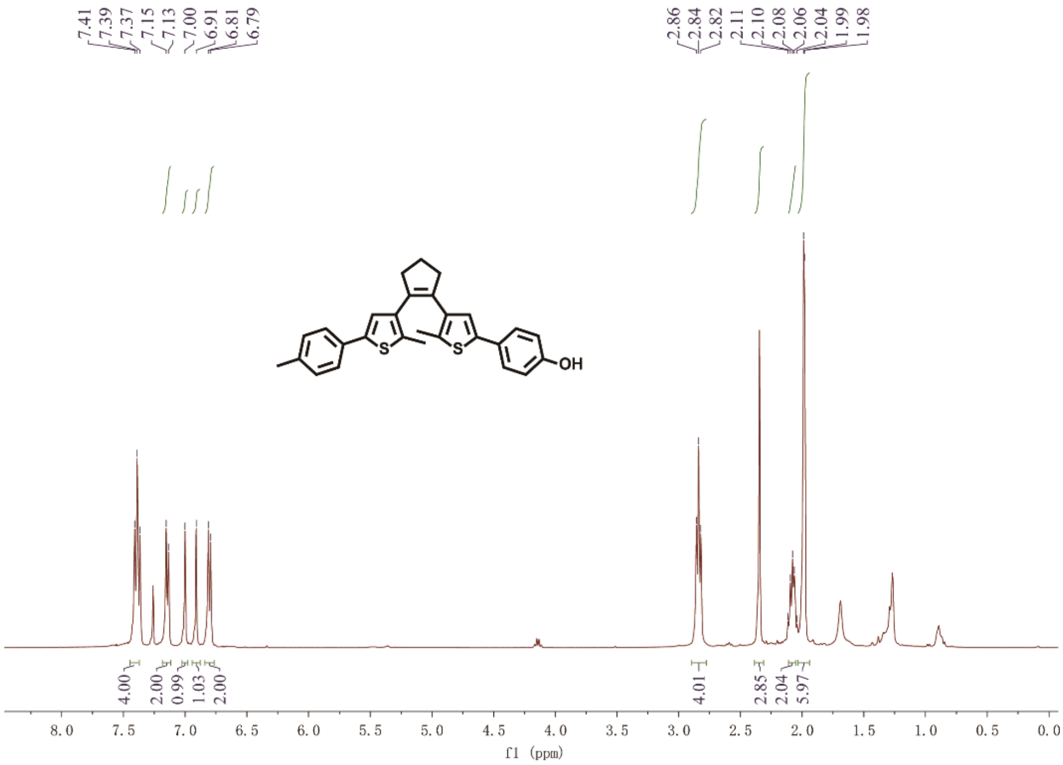

4 (^{13}C NMR, $CDCl_3$)

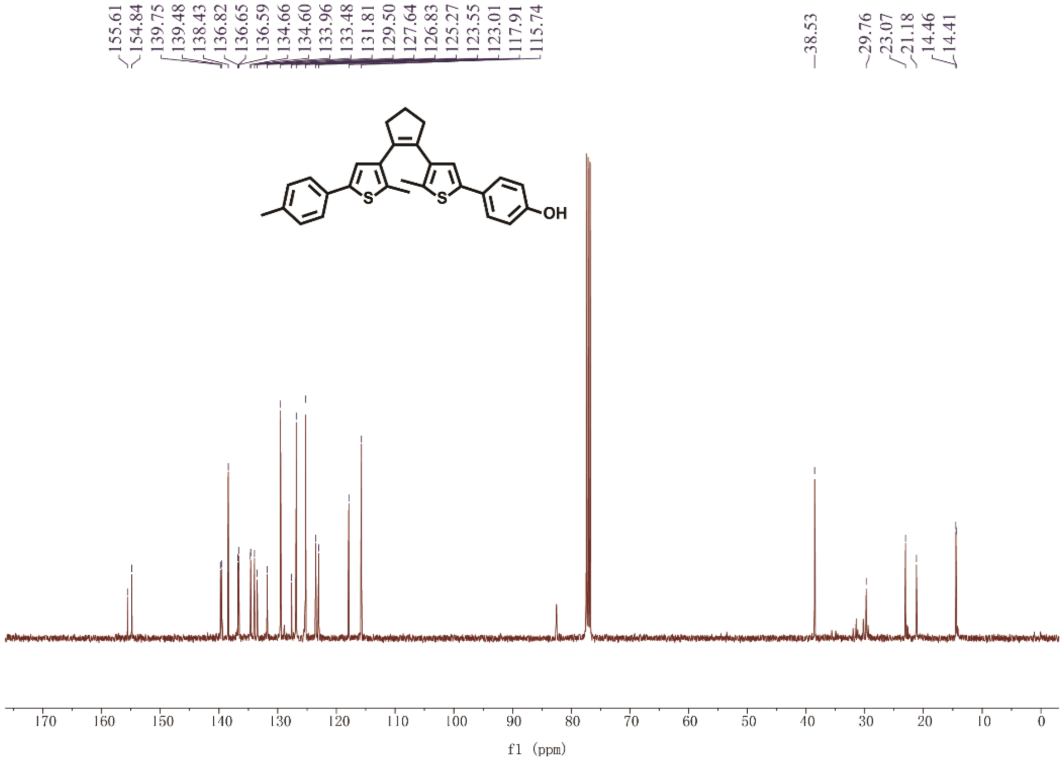

4 (HRMS)

Elemental Composition Report Page 1

Single Mass Analysis
Tolerance = 5.0 mDa / DBE: min = -1.5, max = 50.0
Element prediction: Off
Number of isotope peaks used for i-FIT = 2

Monoisotopic Mass, Even Electron Ions
10 formula(e) evaluated with 1 results within limits (up to 50 best isotopic matches for each mass)
Elements Used:
C: 0-28 H: 0-99 O: 0-1 S: 0-2
H-TIAN
TH-ZZW-091 79 (0.894) Cm (78:81) 1: TOF MS ES- 1.42e+003

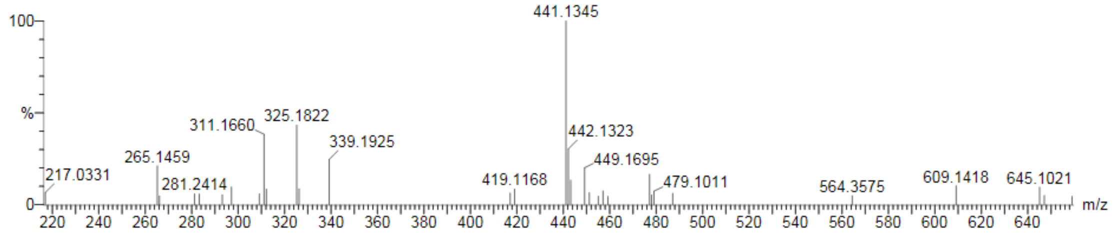

Minimum:				-1.5			
Maximum:		5.0	30.0	50.0			
Mass	Calc. Mass	mDa	PPM	DBE	i-FIT	i-FIT (Norm)	Formula
441.1345	441.1347	-0.2	-0.5	16.5	11.3	0.0	C28 H25 O S2

DAE-1o (^1H NMR, $CDCl_3$)

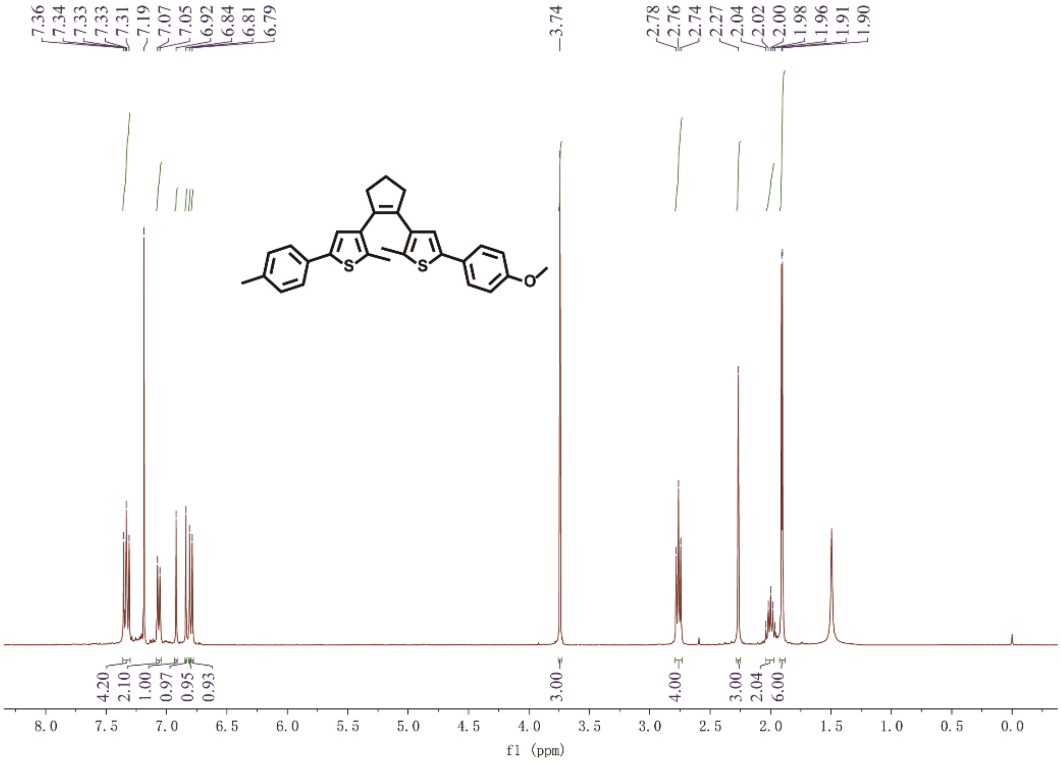

DAE-1o (^{13}C NMR, $CDCl_3$)

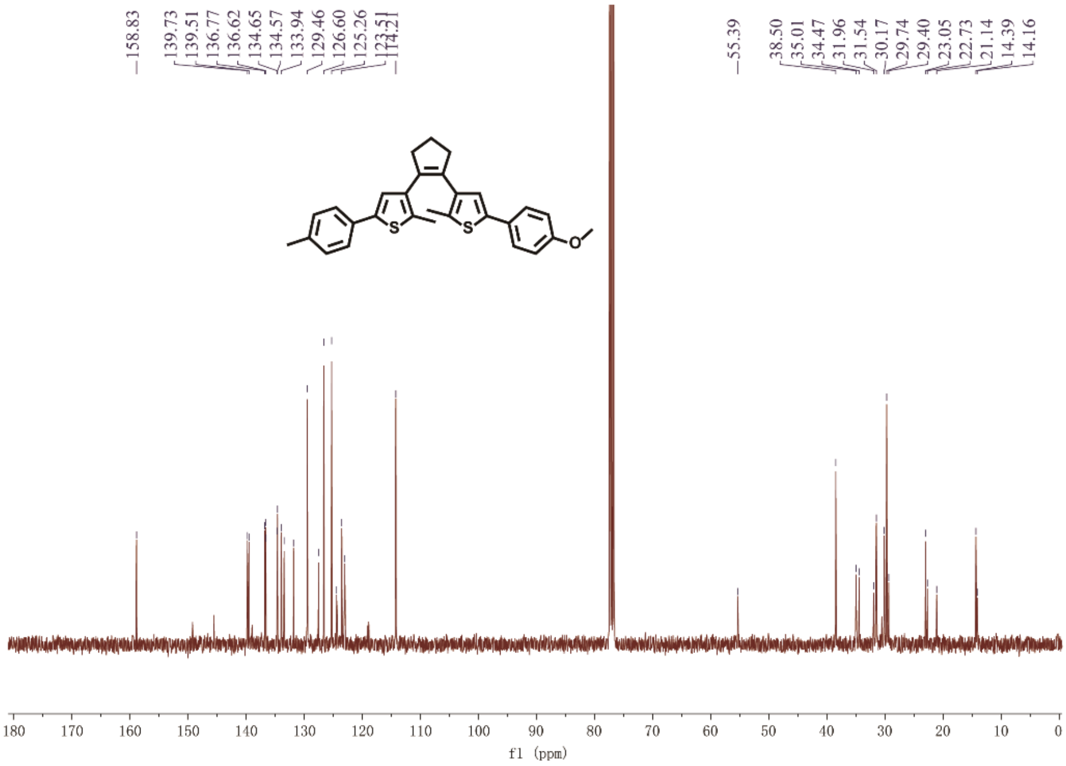

DAE-1o (HRMS)

Elemental Composition Report **Page 1**

Single Mass Analysis
Tolerance = 5.0 PPM / DBE: min = -1.5, max = 50.0
Element prediction: Off
Number of isotope peaks used for i-FIT = 2

Monoisotopic Mass, Even Electron Ions
16 formula(e) evaluated with 1 results within limits (up to 50 closest results for each mass)
Elements Used:
C: 0-29 H: 0-99 O: 0-1 Na: 0-1 S: 0-2
H-TIAN
TH-WWH-002 355 (4.079) Cm (354:359)

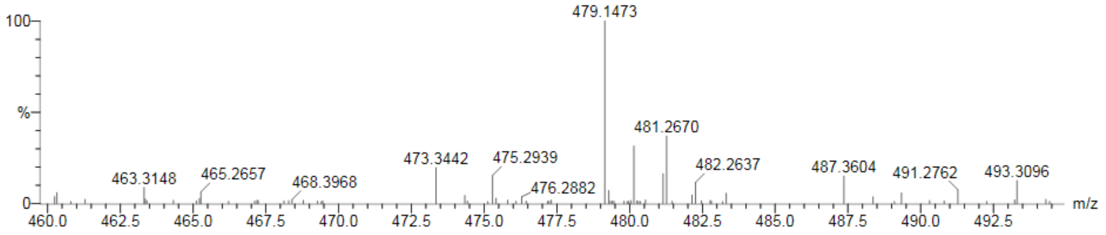

Minimum:				-1.5			
Maximum:		5.0	5.0	50.0			
Mass	Calc. Mass	mDa	PPM	DBE	i-FIT	i-FIT (Norm)	Formula
479.1473	479.1479	-0.6	-1.3	15.5	86.0	0.0	C29 H28 O Na S2

5 (^{1}H NMR, $CDCl_3$)

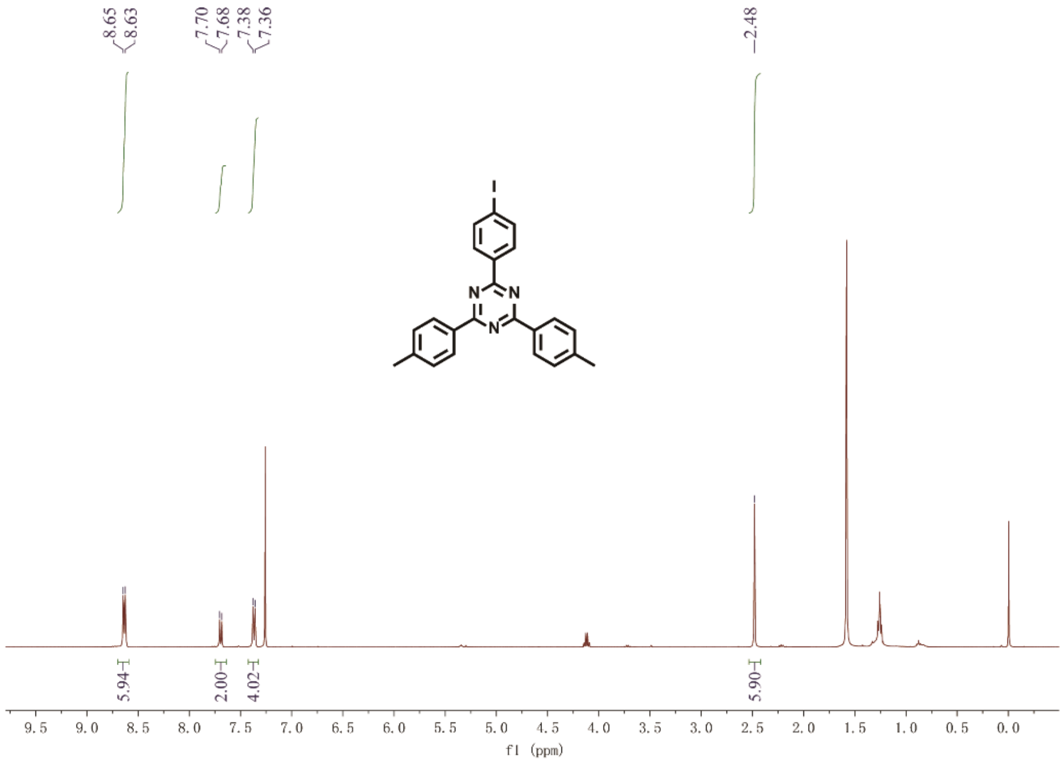

5 (^{13}C NMR, $CDCl_3$)

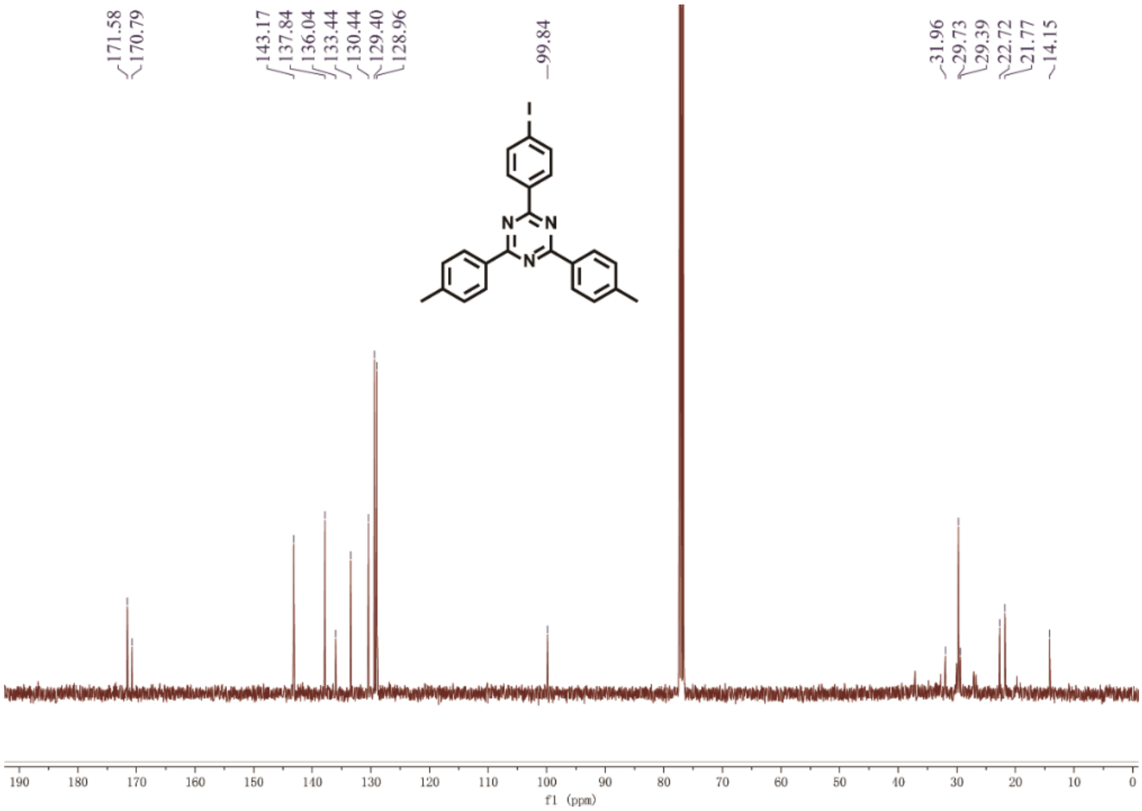

5 (HRMS)

Elemental Composition Report **Page 1**

Single Mass Analysis
Tolerance = 5.0 mDa / DBE: min = -1.5, max = 50.0
Element prediction: Off
Number of isotope peaks used for i-FIT = 2

Monoisotopic Mass, Even Electron Ions
6 formula(e) evaluated with 1 results within limits (up to 50 best isotopic matches for each mass)
Elements Used:
C: 0-23 H: 0-19 N: 0-3 I: 0-1
H-TIAN
TH-ZZW-087 86 (0.996) Cm (83:93)

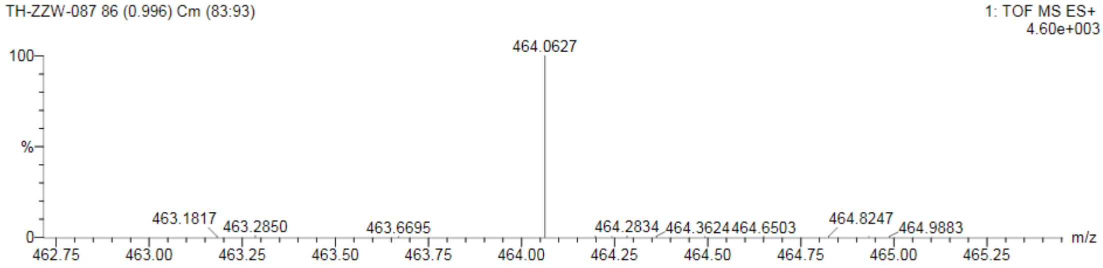

Minimum:				-1.5			
Maximum:		5.0	30.0	50.0			
Mass	Calc. Mass	mDa	PPM	DBE	i-FIT	i-FIT (Norm)	Formula
464.0627	464.0624	0.3	0.6	15.5	76.5	0.0	C23 H19 N3 I

6 (^{1}H NMR, $CDCl_3$)

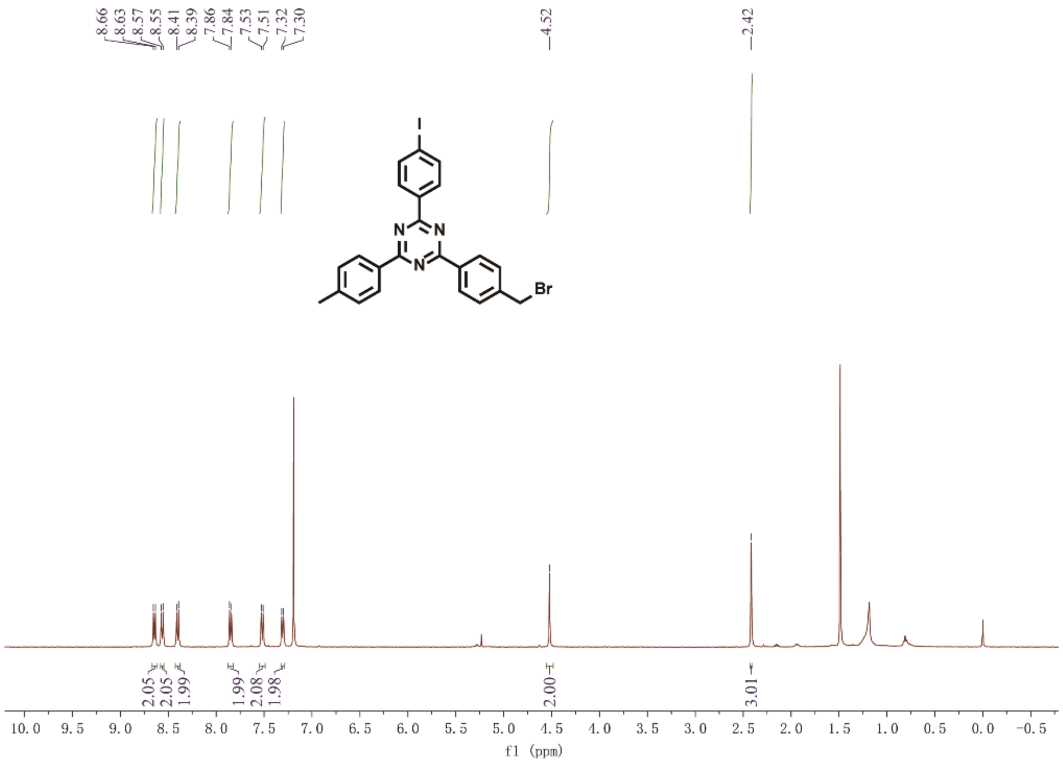

6 (^{13}C NMR, $CDCl_3$)

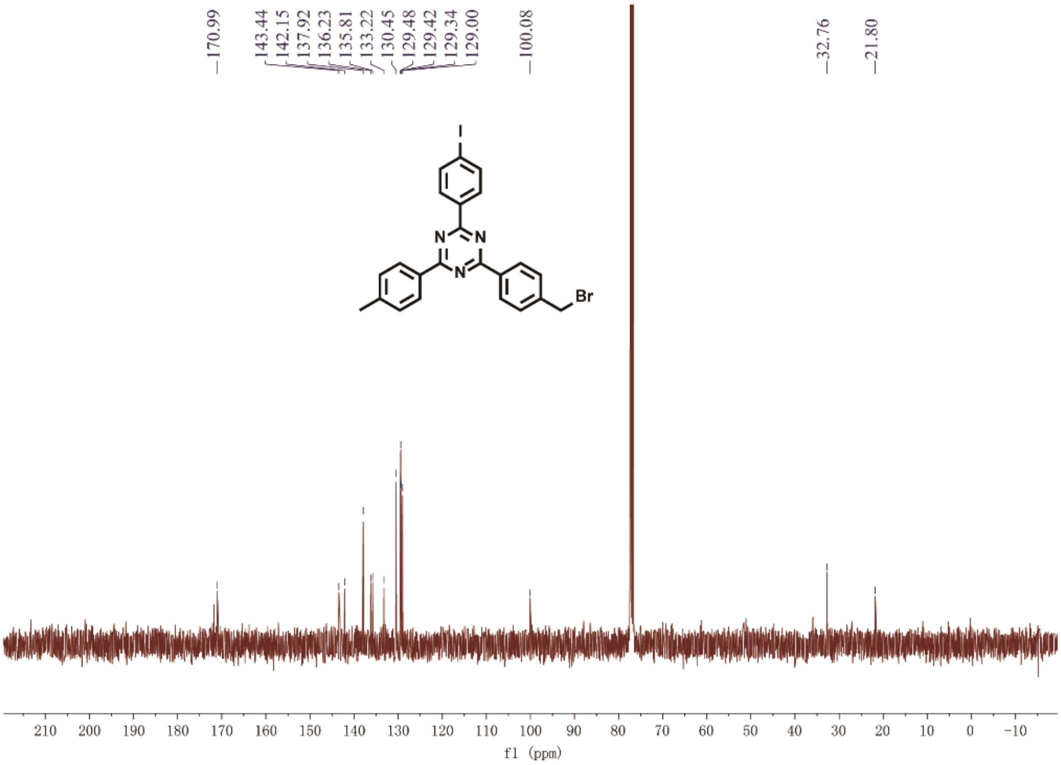

6 (HRMS)

Elemental Composition Report **Page 1**

Single Mass Analysis
Tolerance = 5.0 mDa / DBE: min = -1.5, max = 50.0
Element prediction: Off
Number of isotope peaks used for i-FIT = 2

Monoisotopic Mass, Even Electron Ions
14 formula(e) evaluated with 1 results within limits (up to 50 best isotopic matches for each mass)
Elements Used:
C: 0-23 H: 0-19 N: 0-3 Br: 0-1 I: 0-1

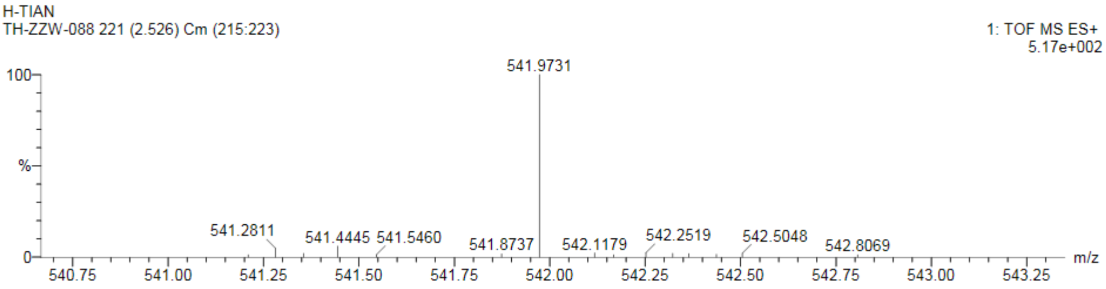

Minimum:				-1.5			
Maximum:		5.0	30.0	50.0			
Mass	Calc. Mass	mDa	PPM	DBE	i-FIT	i-FIT (Norm)	Formula
541.9731	541.9729	0.2	0.4	15.5	55.5	0.0	C23 H18 N3 Br I

7 (^{1}H NMR, $CDCl_3$)

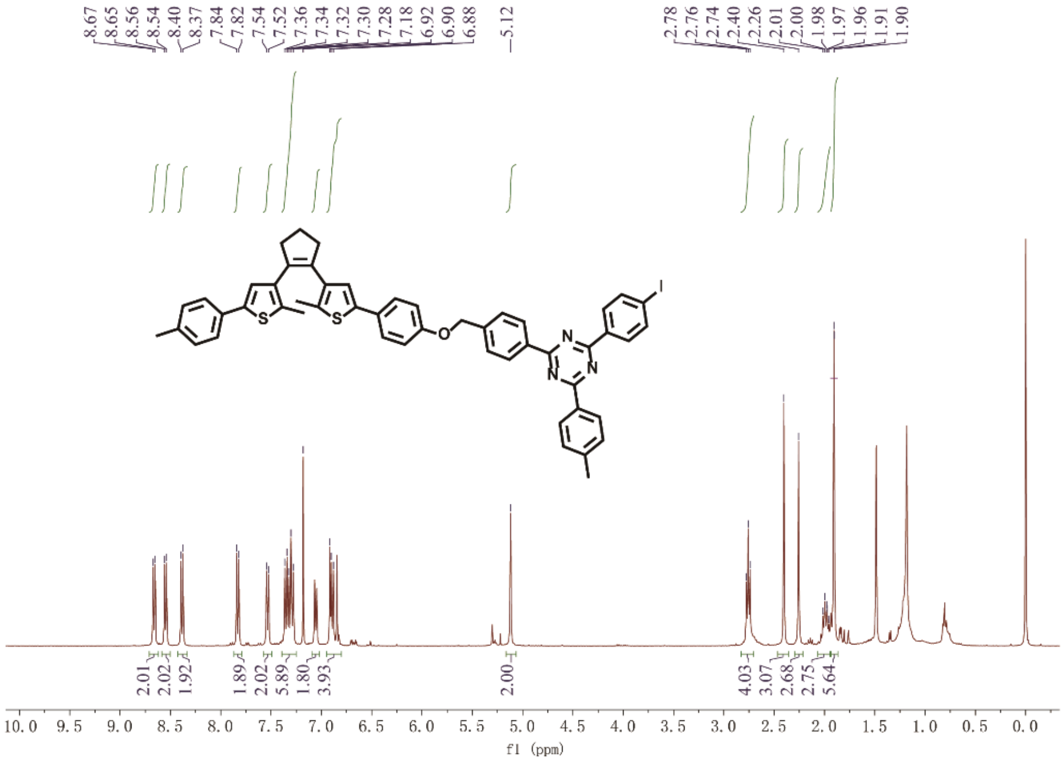

7 (^{13}C NMR, $CDCl_3$)

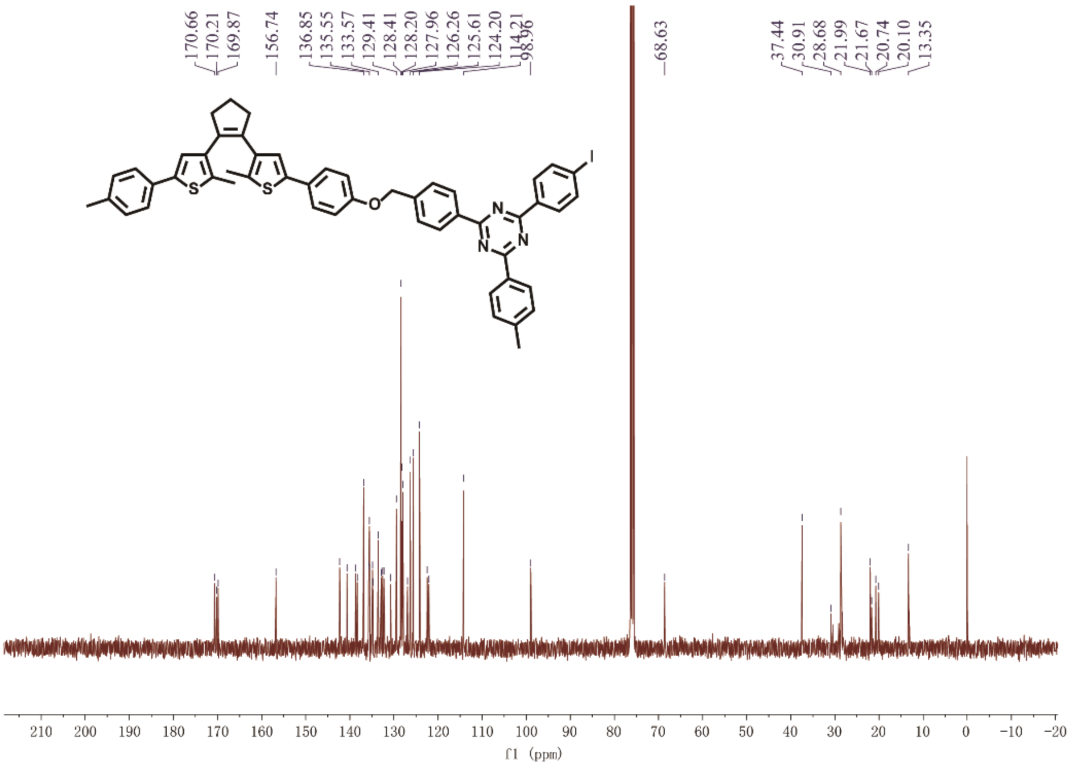

7 (HRMS)

Elemental Composition Report **Page 1**

Single Mass Analysis
Tolerance = 10.0 PPM / DBE: min = -1.5, max = 50.0
Element prediction: Off
Number of isotope peaks used for i-FIT = 2

Monoisotopic Mass, Even Electron Ions
35 formula(e) evaluated with 1 results within limits (up to 50 closest results for each mass)
Elements Used:
C: 21-51 H: 0-50 N: 0-3 O: 0-1 S: 0-2 I: 0-1

H-TIAN
TH-ZZW-003 155 (1.767) Cm (150:155) 1: TOF MS ES+ 9.00e+003

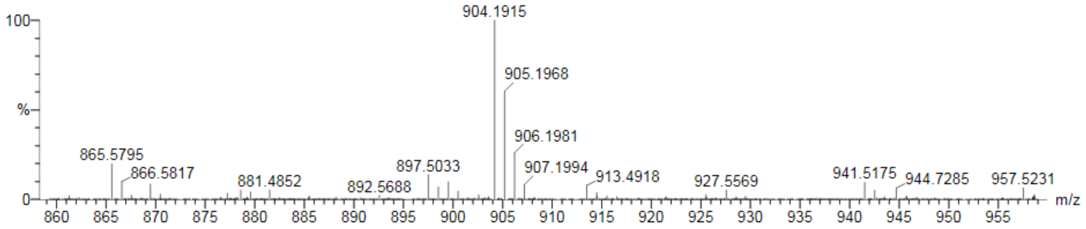

Minimum:				-1.5			
Maximum:		5.0	10.0	50.0			
Mass	Calc. Mass	mDa	PPM	DBE	i-FIT	i-FIT (Norm)	Formula
904.1915	904.1892	2.3	2.5	31.5	78.9	0.0	C51 H43 N3 O S2 I

DT (^{1}H NMR, $CDCl_3$)

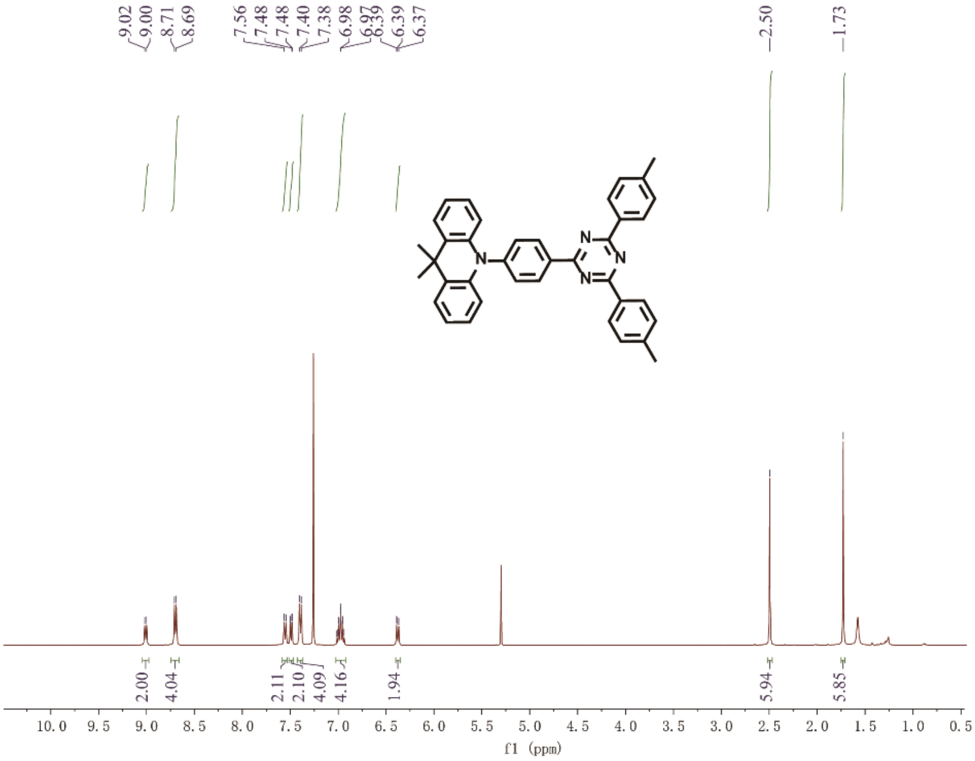

DT (^{13}C NMR, $CDCl_3$)

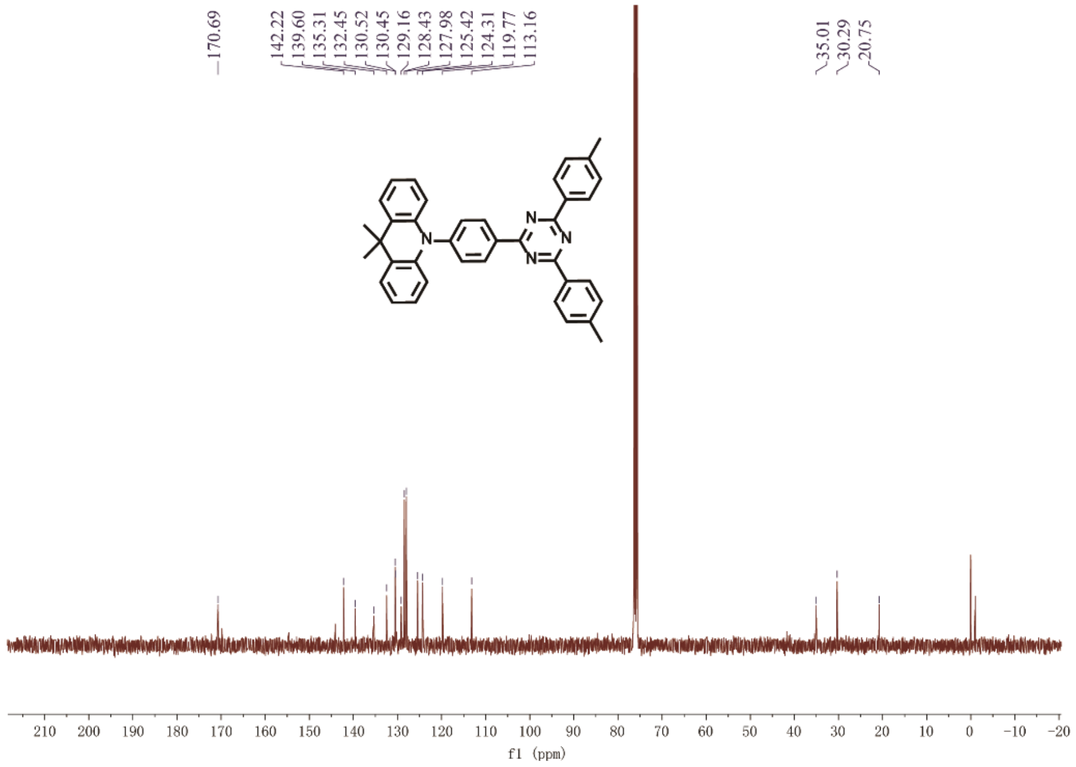

DT (HRMS)

Elemental Composition Report **Page 1**

Single Mass Analysis
Tolerance = 5.0 PPM / DBE: min = -1.5, max = 50.0
Element prediction: Off
Number of isotope peaks used for i-FIT = 2

Monoisotopic Mass, Even Electron Ions
15 formula(e) evaluated with 1 results within limits (up to 50 closest results for each mass)
Elements Used:
C: 0-38 H: 0-99 N: 0-4
H-TIAN
TH-ZZW-090 111 (1.265) Cm (111:113) 1: TOF MS ES+ 2.02e+004

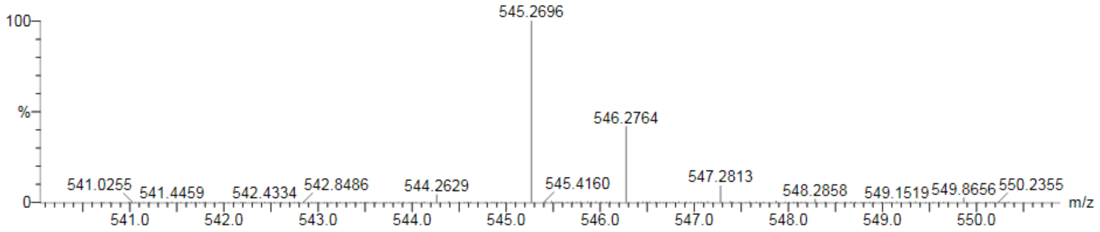

Minimum:				-1.5			
Maximum:		5.0	5.0	50.0			
Mass	Calc. Mass	mDa	PPM	DBE	i-FIT	i-FIT (Norm)	Formula
545.2696	545.2705	-0.9	-1.7	24.5	179.6	0.0	C38 H33 N4

DAE-o-DT (^{1}H NMR, $CDCl_3$)

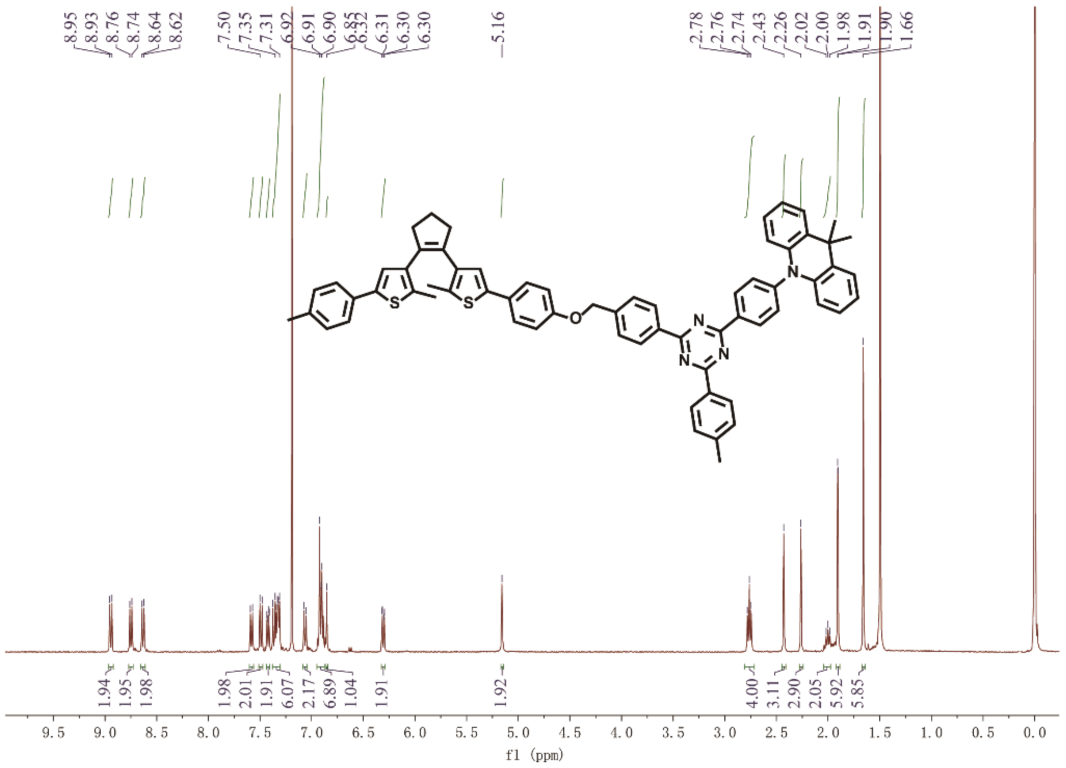

DAE-o-DT (^{13}C NMR, $CDCl_3$)

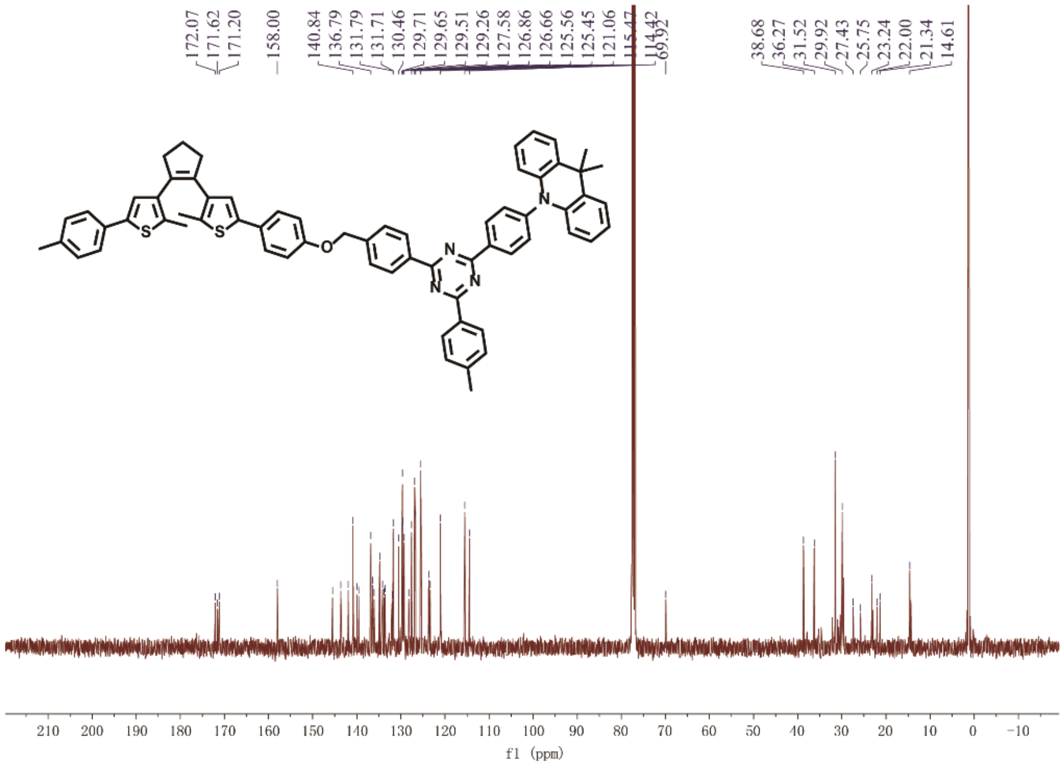

DAE-o-DT (HRMS)

Elemental Composition Report **Page 1**

Single Mass Analysis
Tolerance = 5.0 PPM / DBE: min = -1.5, max = 50.0
Element prediction: Off
Number of isotope peaks used for i-FIT = 2

Monoisotopic Mass, Even Electron Ions
26 formula(e) evaluated with 1 results within limits (up to 50 closest results for each mass)
Elements Used:
C: 0-66 H: 0-99 N: 0-4 O: 0-1 S: 0-2
H-TIAN
TH-ZZW-205 65 (0.729) Cm (63:65) 1: TOF MS ES+ 2.44e+003

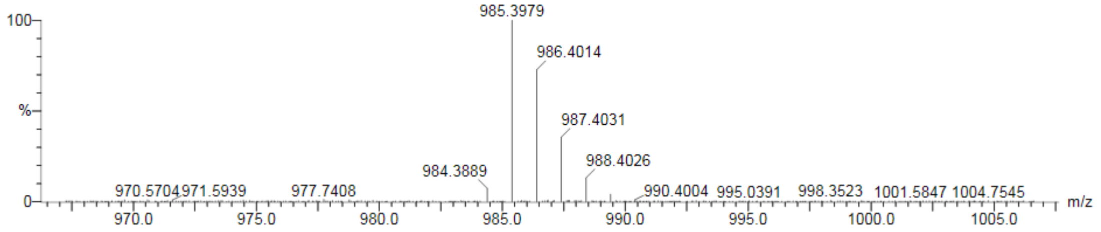

Minimum:				-1.5			
Maximum:		5.0	5.0	50.0			
Mass	Calc. Mass	mDa	PPM	DBE	i-FIT	i-FIT (Norm)	Formula
985.3979	985.3974	0.5	0.5	40.5	58.9	0.0	C66 H57 N4 O S2

8 (^{1}H NMR, $CDCl_3$)

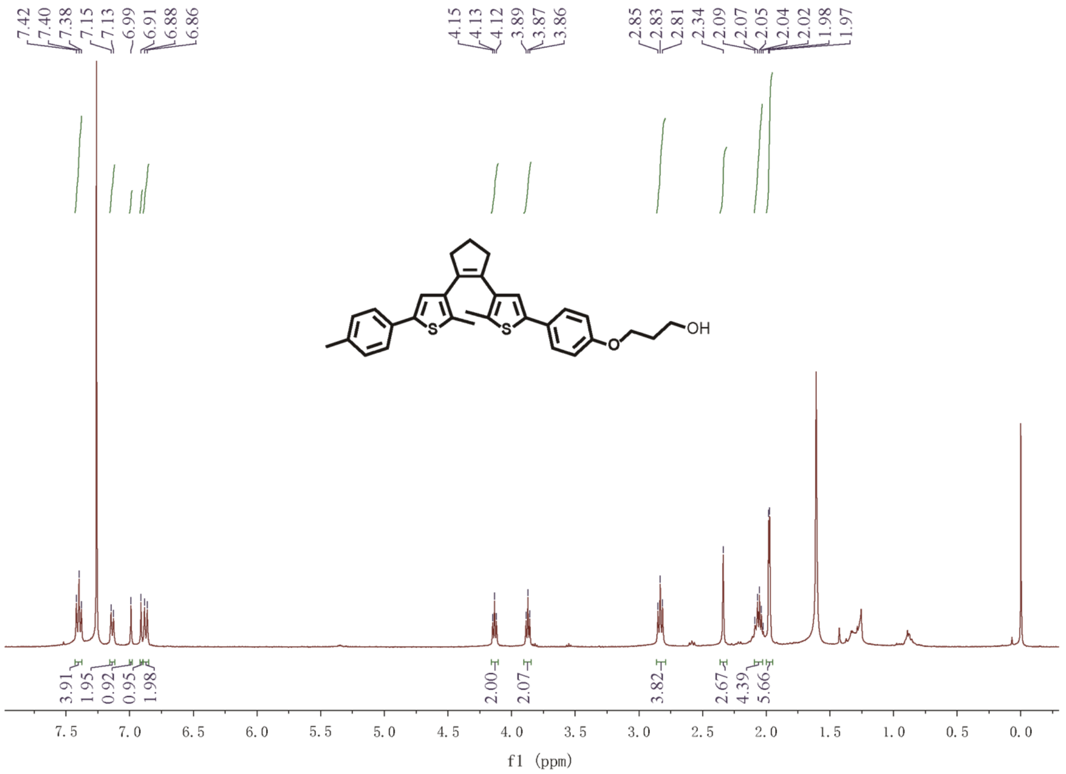

8 (HRMS)

Elemental Composition Report **Page 1**

Single Mass Analysis
Tolerance = 5.0 PPM / DBE: min = -1.5, max = 50.0
Element prediction: Off
Number of isotope peaks used for i-FIT = 2

Monoisotopic Mass, Even Electron Ions
52 formula(e) evaluated with 1 results within limits (up to 50 best isotopic matches for each mass)
Elements Used:
C: 31-31 H: 0-50 O: 0-6 S: 0-2 Na: 0-2

JJ-ZHANG
TH-WWH-014 6 (0.057) Cm (6)

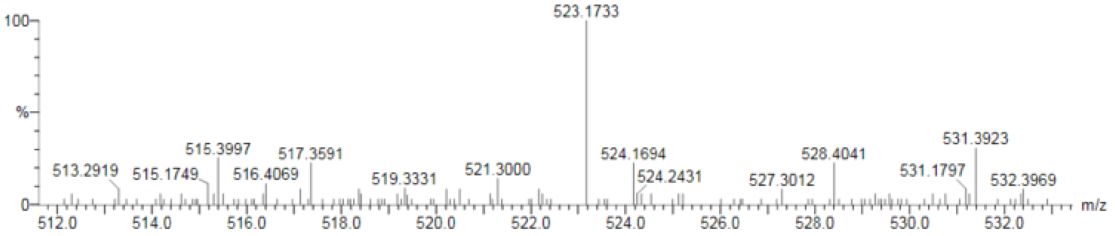

		mDa	PPM	DBE	i-FIT	i-FIT (Norm)	Formula
Minimum:				-1.5			
Maximum:		5.0	5.0	50.0			
Mass	Calc. Mass	mDa	PPM	DBE	i-FIT	i-FIT (Norm)	Formula
523.1733	523.1741	-0.8	-1.5	15.5	20.2	0.0	C31 H32 O2 S2 Na

9 (^{1}H NMR, $CDCl_3$)

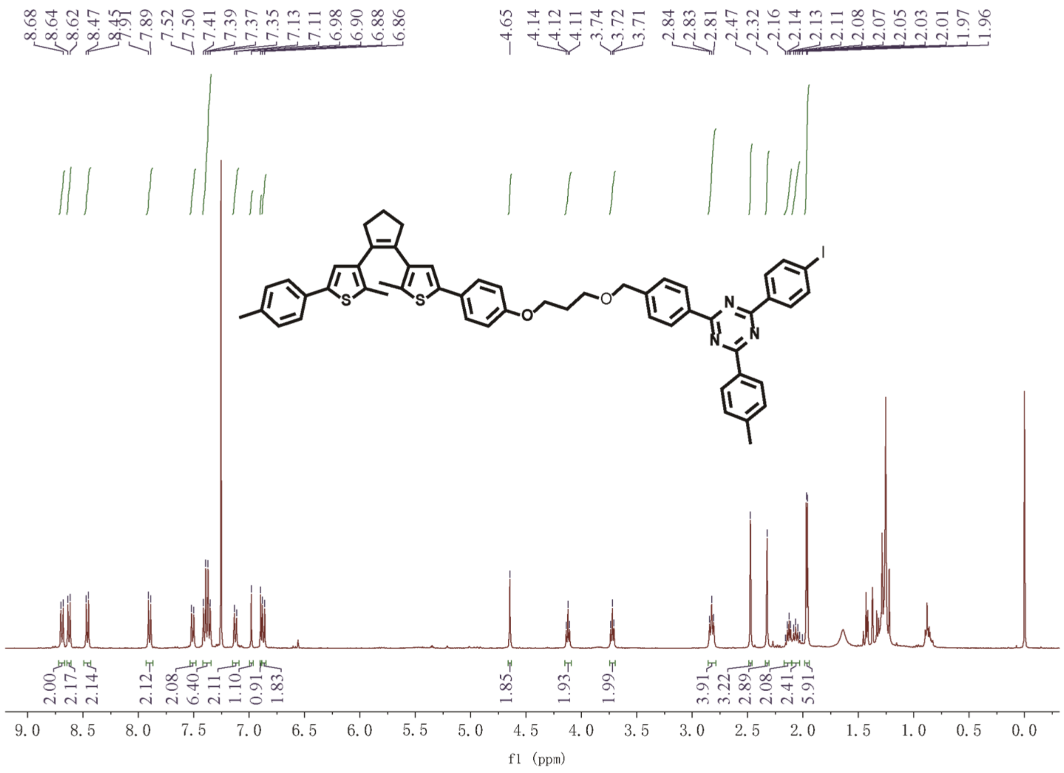

9 (HRMS)

Elemental Composition Report Page 1

Single Mass Analysis
Tolerance = 5.0 PPM / DBE: min = -1.5, max = 50.0
Element prediction: Off
Number of isotope peaks used for i-FIT = 2

Monoisotopic Mass, Even Electron Ions
277 formula(e) evaluated with 1 results within limits (up to 50 best isotopic matches for each mass)
Elements Used:
C: 0-54 H: 0-48 N: 0-3 O: 0-2 S: 0-2 Br: 0-1 I: 0-1 Na: 0-1

JJ-ZHANG
ZJ-WWH-001 22 (0.241) Cm (22:25)
1: TOF MS ES+
1.34e+002

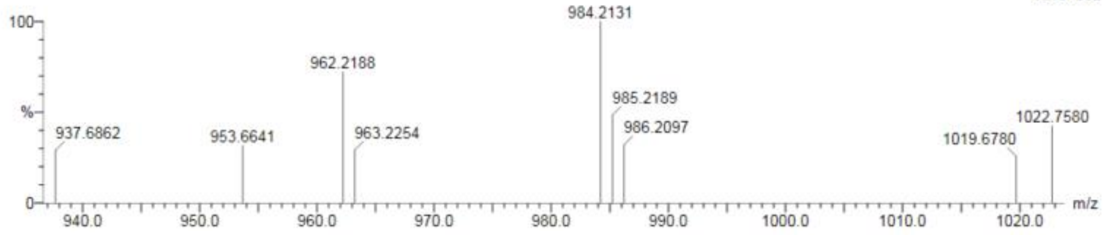

Mass	Calc. Mass	mDa	PPM	DBE	i-FIT	i-FIT (Norm)	Formula
Minimum:				-1.5			
Maximum:		5.0	5.0	50.0			
984.2131	984.2130	0.1	0.1	31.5	10.2	0.0	C54 H48 N3 O2 S2 I Na

DAE-3C-DT (^{1}H NMR, $CDCl_3$)

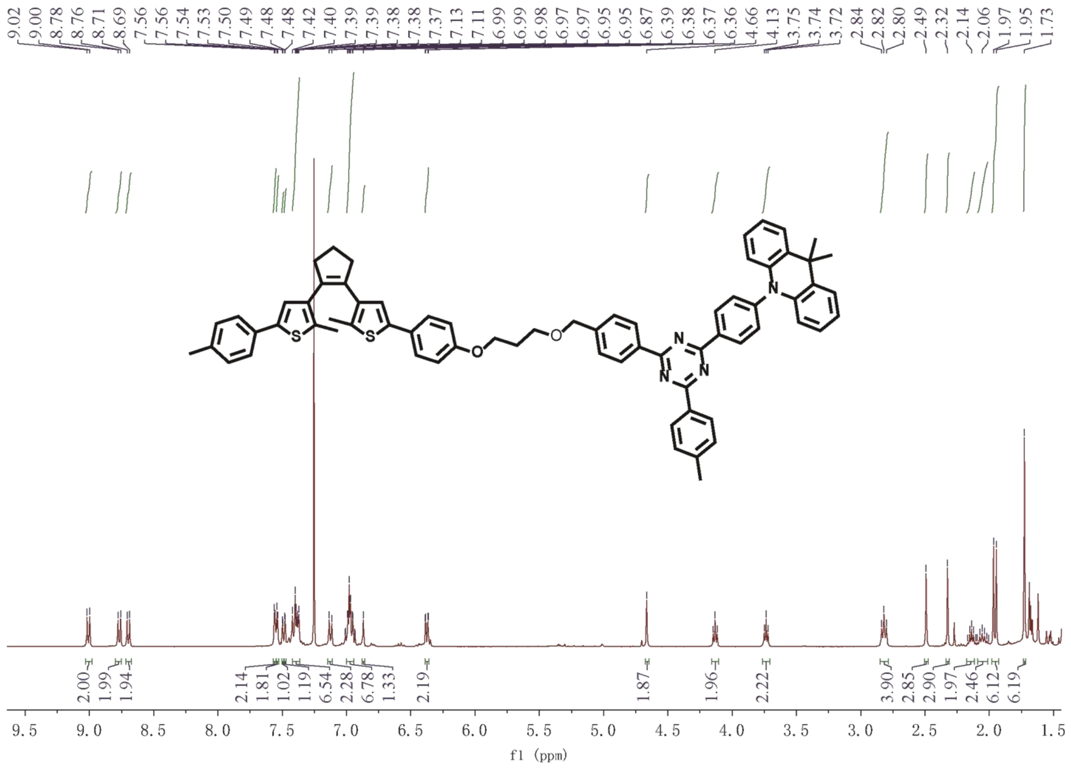

DAE-3C-DT (HRMS)

Elemental Composition Report **Page 1**

Single Mass Analysis
Tolerance = 5.0 PPM / DBE: min = -1.5, max = 50.0
Element prediction: Off
Number of isotope peaks used for i-FIT = 2

Monoisotopic Mass, Even Electron Ions
20 formula(e) evaluated with 1 results within limits (up to 50 best isotopic matches for each mass)
Elements Used:
C: 0-69 H: 0-63 N: 0-4 O: 0-2 S: 0-2
H-TIAN
TH-WWH-013 70 (0.794) Cm (69:73)

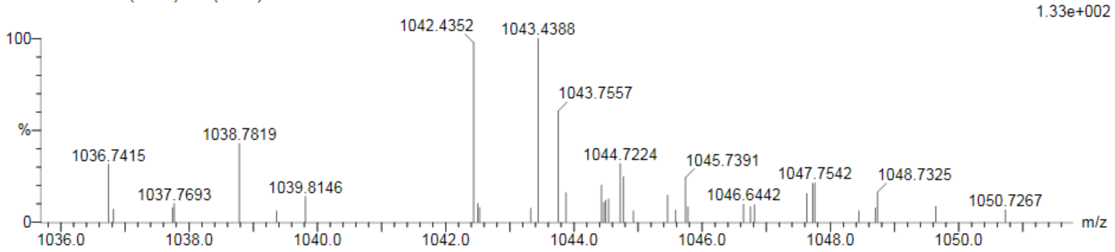

Minimum:				-1.5			
Maximum:		5.0	5.0	50.0			
Mass	Calc. Mass	mDa	PPM	DBE	i-FIT	i-FIT (Norm)	Formula
1043.4388	1043.4392	-0.4	-0.4	40.5	65.6	0.0	C69 H63 N4 O2 S2

Supplementary References

1. Hehre, W. J., Ditchfield, R. & Pople, J. A. Self-consistent molecular orbital methods. XII. Further extensions of Gaussian-type basis sets for use in molecular orbital studies of organic molecules. *J. Chem. Phys.* **56**, 2257-2261 (1972).
2. Yanai, T., Tew, D. & Handy, N. A new hybrid exchange-correlation functional using the Coulomb-attenuating method (CAM-B3LYP). *Chem. Phys. Lett.* **393**, 51-57 (2004).
3. Krishnan, R., Binkley, J. S., Seeger, R. & Pople, J. A. Self-consistent molecular orbital methods. XX. A basis set for correlated wave functions. *J. Chem. Phys*. **72**, 650-654 (1980).
4. Frisch, M. J. et al. *Gaussian 09, revision D. 01*. (2009).
5. Hatchard, C. G. & Parker, C. A. *Proc. Roy. Soc. London, Ser. A* **235**, 518-536 (1956).
6. Montalti, M., Credi, A., Prodi, L. & Gandolfi, M. T. *Handbook of Photochemistry*, Page 601-616. CRC Press (Taylor & Francis Group) (2006).
7. Pijper, T. C. et al. Position and orientation control of a photo-and electrochromic dithienylethene using a tripodal anchor on gold surface. *J. Phys. Chem. C* **119**, 3648-3657 (2015).
